# RNA N^6^-methyladenosine modification mediates downregulation of NR4A1 to facilitate malignancy of cervical cancer

**DOI:** 10.1186/s13578-022-00937-w

**Published:** 2022-12-25

**Authors:** Tao Yu, Fuxia Wu, Yan Jia, Xue Zhang, Xiaozhen Qi, Zeyuan Jin, Tongxin Hao, Jianing Zhao, Ziyu Liu, Chaokun Wang, Minmin Niu, Qin Yue, Min Li, Yankun Liu

**Affiliations:** 1 Department of Pathogen Biology, School of Basic Medical Sciences, Tianjin Medical University, Tianjin, 300070 China; 2 Department of Molecular Diagnosis, Tangshan People’s Hospital, Tangshan, 063001 China; 3 Department of Integrative Chinese and Western Medicine, School of Basic Medical Sciences, Tianjin Medical University, Tianjin, 300070 China

**Keywords:** RNA N^6^-methyladenosine, METTL3, YTHDF2, NR4A1, Cervical cancer, AKT1

## Abstract

**Background:**

N^6^-methyladenosine is the most abundant eukaryotic mRNA modification and alters a wide range of cellular processes in cancer. Therefore, defining the molecular details are critical for understanding the regulatory mechanism of m^6^A modification.

**Results:**

We found that METTL3, a core m^6^A methyltransferase component, is upregulated and functions as an oncogene in cervical cancer. Mechanistically, METTL3 induces the degradation of m^6^A-modified transcripts of NR4A1 though YTHDF2-DDX6 pathway. In addition, NR4A1 overexpression attenuates the malignant progression through recruiting the LSD1/HDAC1/CoREST transcriptional repression complex to AKT1 promoter.

**Conclusions:**

Our findings reveal that m^6^A regulates cervical cancer cellular progression through manipulating NR4A1 pathway.

**Supplementary Information:**

The online version contains supplementary material available at 10.1186/s13578-022-00937-w.

## Background

N^6^-methyladenosine (m^6^A) has been identified as the most abundant internal modification present in the messenger RNA (mRNA) of eukaryotes [1]. As a reversible and dynamic epigenetic modification in mRNA, m^6^A modification has been found to be a widespread regulatory mechanism for controlling gene function in diverse physiological and pathological conditions [2]. In most cases, m^6^A modification sites have a canonical consensus motif DRACH (D = G, A, or U; R = G or A; H = A, C, or U), which are enriched in the coding sequence (CDS), 3’ untranslated region (3’ UTR) and near stop codons in mRNAs [3]. The m^6^A modification is catalyzed by its dedicated methyltransferases complex (“writers”), demethylases(“erasers”) and binding proteins (“readers”) that respectively install, remove and recognize the m^6^A-modified mRNAs. The methyltransferase complex consists of METTL3, METTL14, WATP and other accessory subunits. Within this complex, METTL3 acts as the sole catalytic subunit that binds to the methyl-group donor S-adenosylmethionine (SAM) and transfers a methyl group to target RNA, while METTL14 serves as an essential component for binding the substrate RNA and stabilizing the conformation of METTL3 [4]. WTAP shows its m^6^A function by recruiting and maintaining METTL3-METTL14 complex in nuclear speckles to efficiently methylate mRNA [5]. The m^6^A modification is removed by demethylase FTO and ALKBH5 [1, 2], thus maintaining the dynamic m^6^A regulation under physiological and pathological conditions.

The fate and function of m^6^A-modified RNAs are controlled by m^6^A reader proteins, including the YTH protein family (YTHDF1, YTHDF2, YTHDF3, YTHDC1 and YTHDC2), which specifically recognize and bind to m^6^A sites in target RNAs. In most cases, m^6^A reader affects the molecular mechanisms of RNA processing while do not involve the regulation of global changes of m^6^A levels. In YTH protein family, YTHDF1 accelerates the RNA stabilization and translation initiation [6]. YTHDF2 enhances the degradation of m^6^A-modified transcripts [7, 8]. YTHDF3 regulates translation efficiency of its target RNA through interacting with YTHDF1, and induces RNA decay by cooperating with YTHDF2 [9]. These modifications affect several aspects of RNA processing and metabolism through m^6^A-dependent post-transcriptional regulation mechanisms. Prominently, two recent studies have proposed that YTHDF proteins function similarly and cooperate to mediate degradation of m^6^A-modified mRNAs in specific cells [10, 11]. These findings add additional explanation to the functional complexities of YTHDF paralogs model.

Emerging evidences focusing on m^6^A modification indicate that the regulatory enzymes of m^6^A function as critical factors in numerous important tumor biological processes, including cellular metabolism [12], drug resistance [13] and metastasis [14, 15]. Recent studies have reported that METTL3 acts as an oncogenic role by modulating the fate of its modified mRNA via YTHDF2-dependent m^6^A mechanism in prostate cancer [15] and colorectal carcinoma [16]. Although studies have also demonstrated a partial function of m^6^A modification in cervical cancer (CC) [12, 14, 17, 18], our understanding of the regulatory mechanism and how m^6^A regulator proteins synergistically induce specific tumor malignant phenotype is largely unknown.

In this study, METTL3 was found to promote the malignant biological progression in CC cells. Mechanistically, METTL3-mediated m^6^A modification accelerates NR4A1 mRNA degradation via YTHDF2-dependent pathway, thereby alleviating transcriptional repression of ATK and activating the AKT signaling pathway in CC cells. Overall, our findings expand the landscape of m^6^A modification in epitranscriptomic regulation that occurs in CC cells.

## Results

### METTL3 is highly expressed in CC tissue and promotes CC cells progression

**Fig. 1:**
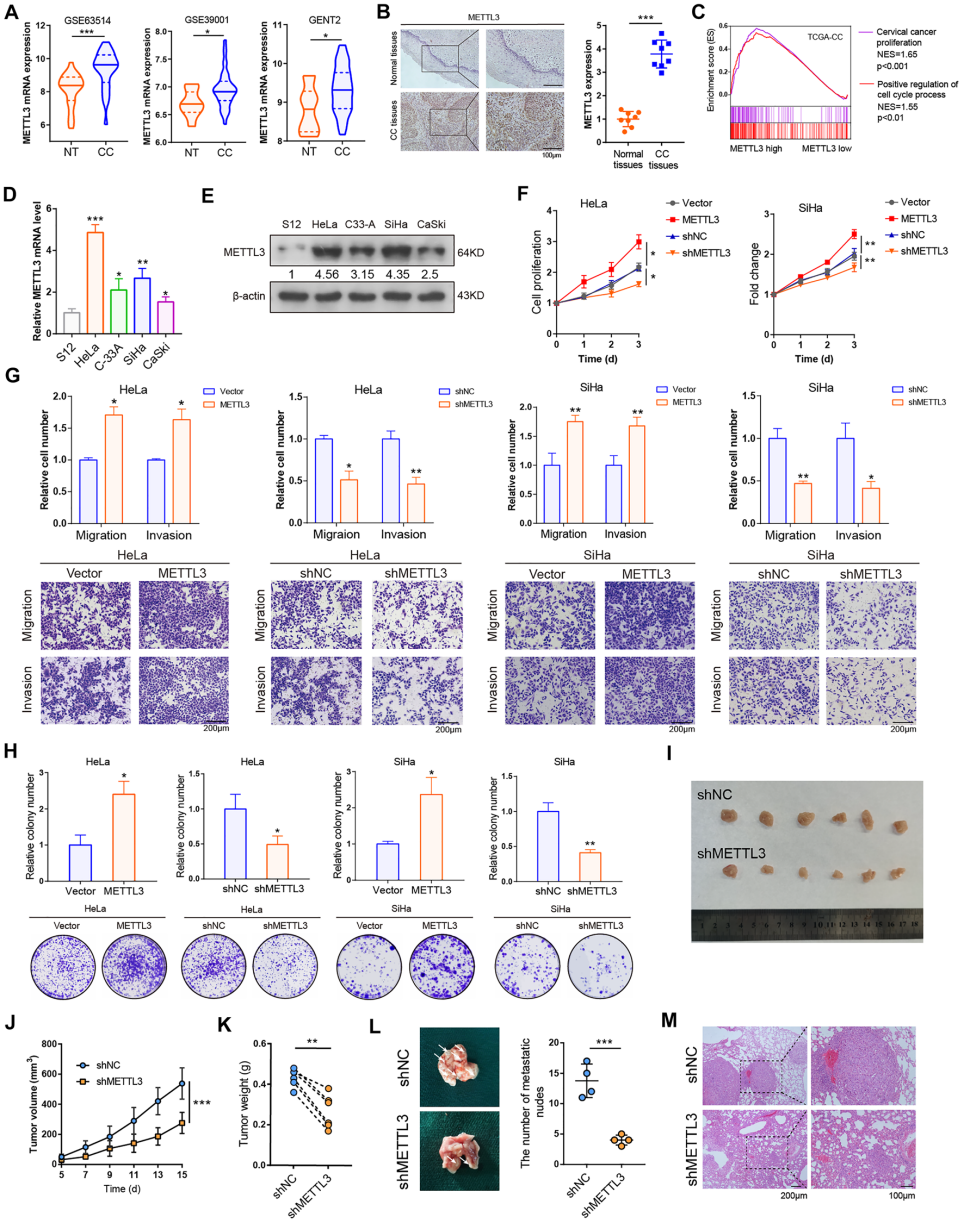
METTL3 plays an oncogenic role in CC. Supplementary Information The mRNA expression of METTL3 in GEO (GSE39001 and GSE63514) and GENT2 CC cohorts. The three lines inside the violin plots are the first quartile, median and third quartile. **B** Representative IHC staining images of METTL3 protein from CC tissues and paired adjacent non-tumor tissues (n = 8, left) and quantitatively analyzed (right). C, GSEA plot of cellular process in TCGA-CC samples. **D**, **E** The mRNA(D) and protein (**E**) levels of METTL3 in CC cell lines (HeLa, C-33A, SiHa and CaSki) and normal cervical epithelial cells (S12). **F** MTT assay displaying the effect of METTL3 on cell proliferation in HeLa and SiHa cells. G, Transwell assays were used to examine the migratory and invasive abilities of HeLa and SiHa cells with overexpression and knockdown of METTL3. **H** Colony formation assay of HeLa and SiHa cells with overexpression and knockdown of METTL3. I-K, Stable METTL3-silenced and control HeLa cells were subcutaneously implanted into mice. The tumor sizes were continuously recorded to draw tumor growth curves (**J**). The tumors were collected at the endpoint of the xenograft models and tumor weights were measured (**K**). **L**, **M** Metastatic tumor foci in lung were quantified (**L**) and confirmed by HE staining (**M**). All experiments were performed at least 3 independent times, and data are presented as means ± SD except where otherwise specified. *P < 0.05, **P < 0.01, ***P < 0.001

To reveal the expression of METTL3 in CC tissues, we first analyzed the available clinical data from TIMER2.0 [19], GENT2 (http://gent2.appex.kr/gent2/) and GEO datasets (GSE39001, GSE63514), and found that METTL3 mRNA levels was highly expressed in CC tissues compared to the normal controls (Fig. 1A; Additional file 1: Fig. S1A). Immunohistochemistry staining (IHC) also showed a significantly increased expression of METTL3 in CC tissues compared to adjacent non-tumor tissues (Fig. 1B). Survival analysis using Kaplan-Meier Plotter database [20] and GEPIA2 database [21] identified that CC patients with high METTL3 levels exhibited a worse disease-free survival (DFS) (Additional file 1: Fig. S1B). The Gene set enrichment analysis (GSEA) [22] results revealed that CC proliferation and cell cycle process were significantly enriched in METTL3-high tissues by using TCGA-CC results (Fig. 1C). In an analysis of a genome-scale CRISPR–Cas9 essentiality screening dataset (https://depmap.org/portal/) [23], we found that METTL3 was an important gene for proliferation and survival of various cancer cells including CC (Additional file 1: Fig. S1C). In addition, METTL3 expression was also assessed in immortalized normal human cervical epithelial cell line S12 and CC cells lines (HeLa, C-33A, SiHa and CaSik) by using western blot assay and RT-qPCR assay. Consistent with the results from CC tissues, METTL3 was significant highly expressed in various CC cell lines compared with S12 cell line. Among these four CC cell lines, HeLa and SiHa cells displayed the highest levels of METTL3 (Fig. 1D, E). Thus, these data imply that METTL3 may be involved in the cellular malignancy of CC.

Then, to determine the role of METTL3 in CC cells, we successfully used gain- and loss-of-function assay in HeLa and SiHa cells by overexpressing METTL3 and knockdown of METTL3 by using specific shRNAs, respectively (Additional file 1: Fig. S1D). As shown in Fig. 1F, MTT assay indicated that enforced expression of METTL3 significantly promoted, whereas knocking down of METTL3 suppressed the cell viability in CC cells. Indeed, upregulation of METTL3 significantly elevated CC cells migration and invasion, and attenuation of METTL3 expression apparently impeded the migratory and invasive ability of CC cells (Fig. 1G). The colony formation assay revealed that overexpression of METTL3 promoted, while knock-down of METTL3 reduced the colony formation rate in HeLa and SiHa cells (Fig. 1H). Next, we explored that whether METTL3 drivers the EMT program in CC cells. As shown in Fig. S1E western blot assay revealed that overexpression of METTL3 up-regulated N-cadherin and Vimentin protein as well as decreased the protein levels of E-cadherin, while knockdown of METTL3 caused an opposite result in CC cells. To further confirm the role of METTL3 in CC tumor in vivo, we applied the subcutaneous xenograft mouse models. Knockdown of METTL3 dramatically suppressed the formation, progression and lung metastasis of CC tumors in vivo (Fig. 1I–M). Meanwhile, METTL3 deletion markedly impeded Ki-67 activity in tumor tissues compared to controls (Additional file 1: Fig. S1F). Collectively, these results indicate the critical role of METTL3 in promoting the malignant phenotype of CC cells.

### NR4A1 is identified as the candidate target of METTL3

**Fig. 2:**
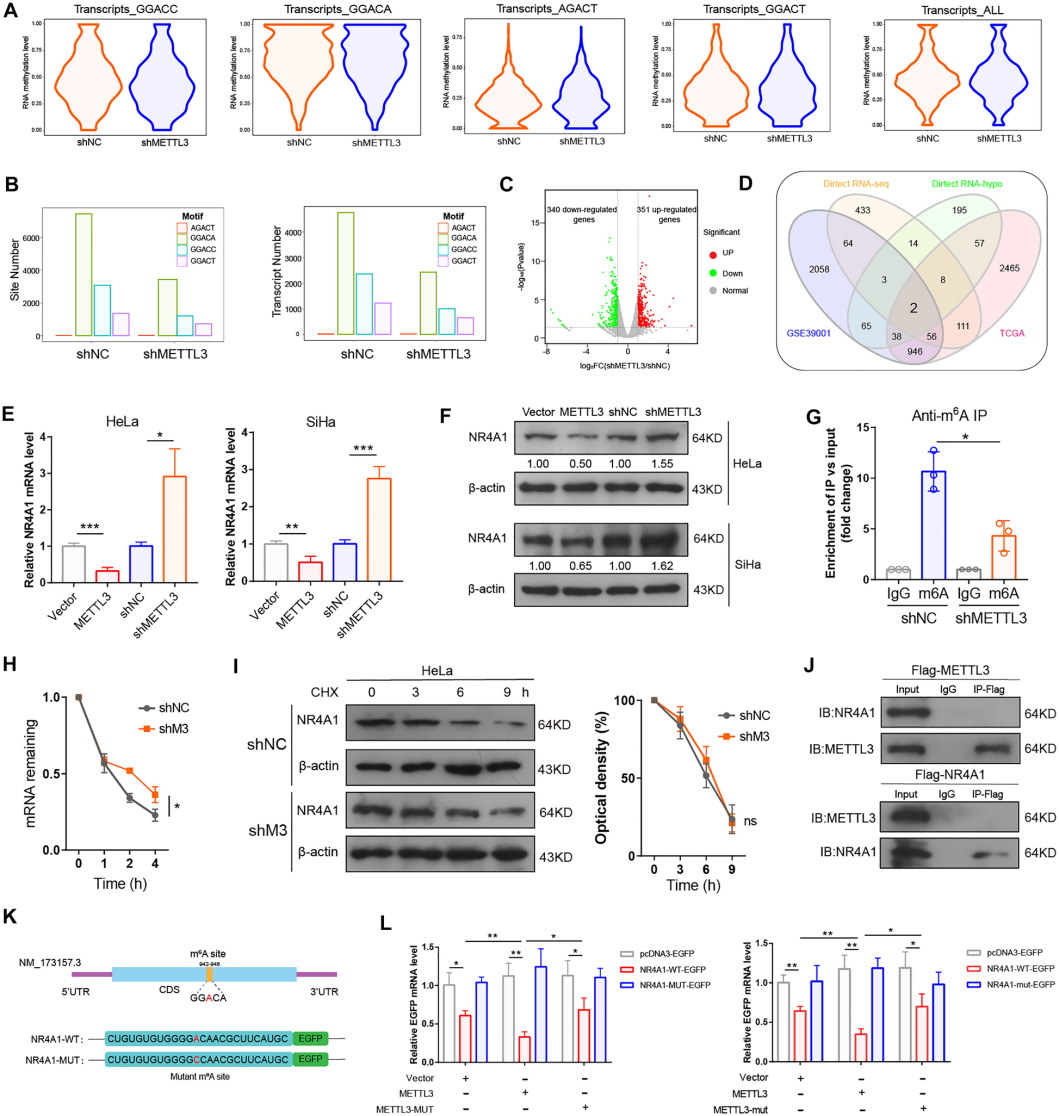
METTL3 inhibits NR4A1 expression via m^6^A-dependent manner. **A**, **B** Direct RNA-seq analysis of the m^6^A abundance and sites in shR-NC and shR-METTL3 HeLa cells. **C** Volcano plot displaying the differentially expressed genes base on direct RNA-seq. **D** Venn diagram showed the candidate target gene from TCGA, GSE39001 and Direct RNA-seq. **E**, **F** RT-qPCR and western blot assays of the mRNA (**E**) and protein (**F**) expression of NR4A1 in CC cells with overexpression and knockdown of METTL3. **G** m^6^A RIP-qPCR analysis of NR4A1 mRNA in control and METTL3-silenced HeLa cells. H, The stability of NR4A1 mRNA in control and METTL3-silenced HeLa cells. Transcription was inhibited by act-D at 5 µg/ml during indicated times. **I** Left: Cells were incubated with 100 µg/ml CHX at the indicated times, the protein stability of NR4A1 was detected by western blot assay. Right: Quantification of protein stability. **J** Co-IP assay for the interactions between METTL3 and NR4A1. **K** Schematic depiction of mutated (GGACA to GGCCA) m^6^A site in CDS region in NR4A1. **L** RT-qPCR analysis showed the expression of EGFP from cells transfected with the indicated plasmids, and normalized to the corresponding β-actin (left) and RFP (right) values. All experiments were performed at least 3 independent times, and data are presented as means ± SD. *P < 0.05, **P < 0.01, ***P < 0.001

To explore the potential mechanism underlying the protumorigenic effect of METTL3-catalyzed m^6^A modification in CC cells, we performed direct RNA-seq (direct sequencing of native RNA, a third-generation sequencing using Oxford Nanopore technology to detect RNA N^6^-methyladenosine modifications in endogenous transcripts) [24] in shR-METTL3/shR-NC HeLa cells. METTL3 knockdown caused a significant reduction in m^6^A abundance and m^6^A sites in transcripts with different m^6^A motifs (GGACC, GGACA, AGACT, GGACT) (Fig. 2A, B). In addition, 691 differentially expressed genes (340 down-regulated genes and 351 up-regulated genes) and 382 hypomethylated genes in METTL3-silenced HeLa cells group compared with normal control HeLa cells group (Fig. 2C, D; Additional file 2: Table S2). Then, we obtained the overlapped genes in TCGA dataset, GEO dataset (GSE39001) and direct RNA-seq, two genes (NR4A1 and GAPDH) were overlapped in these groups (Fig. 2D). Through a literature review, we found that NR4A1 was reported as a key factor for cancer initiation and progression [25, 26], therefore was selected for the further study. To validate NR4A1 was a candidate target of METTL3, we examined the expression of NR4A1 in METTL3-overexpressing and METTL3-knockdown CC cells by RT-qPCR and western blotting. Overexpression of METTL3 down-regulated both mRNA and protein levels of NR4A1 in CC cells, whereas knockdown of METTL3 increased the expression of endogenous NR4A1 in HeLa cells and SiHa cells (Fig. 2E, F).

As previous work reported, METTL3 depend on its residues 395–398 (DPPW) for selective recognition of m^6^A [27]. We therefore mutated the m^6^A recognition sites converted from DPPW to APPA, which abrogate the specific binding affinity to m^6^A-modified mRNA of METTL3. Then we found that the expression of NR4A1 was not significantly different by transfection with exogenous expression of METTL3 catalytic inactive mutant plasmid (METTL3-D395A/W398A-MUT) in CC cells compared with that in control group (Additional file 1: Fig. S2A, B). m^6^A-RIP-qPCR was then applied to confirm the m^6^A-mediated methylation of NR4A1mRNA. When compared to the IgG group, approximately 11-fold enriched of NR4A1 mRNA was obtained by the reaction to m^6^A-specific antibody in HeLa cells, whereas this enrichment was significantly reduced in shR-METTL3 HeLa cells (Fig. 2G). We then studied the impact of METTL3 on the stability of NR4A1 mRNA in HeLa cells. After treated with actinomycin D (act-D) to block transcription at indicated time points, the stability of NR4A1 mRNA was increased in HeLa cells by METTL3-knockdown (Fig. 2H). We further investigated whether METTL3 can regulate the protein stability of NR4A1. Both sh-NC and shR-METTL3 HeLa cells were treated with protein translation inhibitor cycloheximide (CHX) at indicated time points. The results revealed that the protein stability of NR4A1 had no significant difference between these two groups (Fig. 2I). Co-IP assay demonstrated that METTL3 protein was not interacted with NR4A1 protein (Fig. 2J). All these data suggest that METTL3-mediated m^6^A modification induces NR4A1 mRNA degradation, rather than regulating protein stability or directly post-translation modification in CC cells.

In order to determine the position of m^6^A modification site in NR4A1 mRNA, we firstly analyzed our direct RNA-seq. According to the result, the m^6^A modification site was at CDS region of NR4A1 (Fig. 2K). We then generated an enhanced green fluorescent protein (EGFP) reporter with CDS fragments or mutant CDS fragments of NR4A1 harboring METTL3-mediated m^6^A modification motif and transfected into HeLa cells along with METTL3-WT, METTL3-MUT or the control vector (Fig. 2K). Western blot, RT-qPCR and fluorescence microscopy assays showed that overexpression of METTL3 in HeLa cells reduced the activity of the NR4A1-EGFP report with wild-type m^6^A modification site, but did not affect the levels of the NR4A1-EGFP reporter with m^6^A mutation site (Fig. 2L; Additional file 1: Fig. S2C–E). In addition, overexpression of catalytic mutant METTL3 had no significant effect on regulating the EGFP expression of wild-type NR4A1-EGFP and m^6^A mutation NR4A1-EGFP compared to the control vector (Fig. 2L; Additional file 1: Fig. S2C–E).

### Overexpression of NR4A1 abolishes the aggressive phenotypes induced by METTL3 in CC cells

**Fig. 3:**
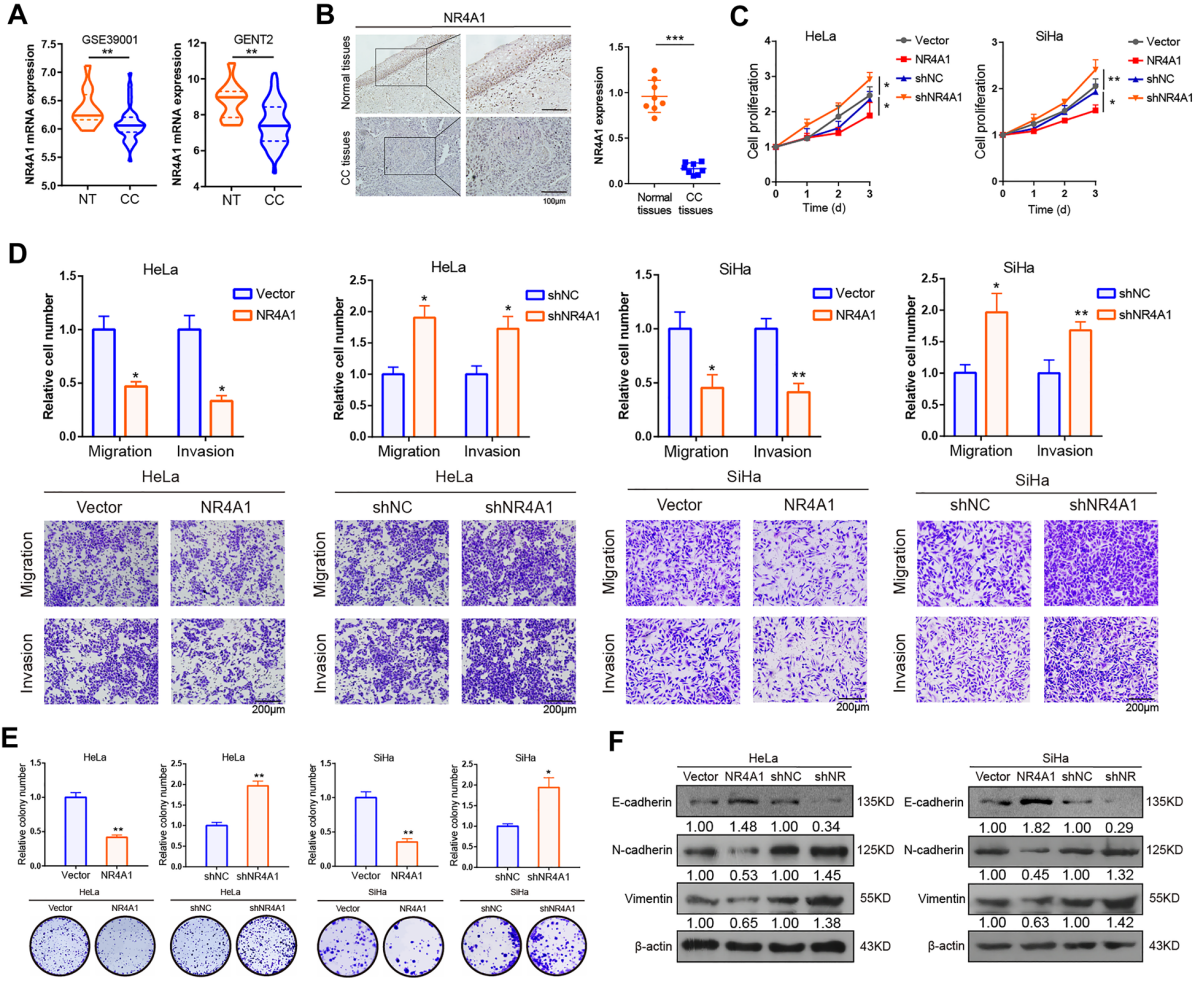
NR4A1 suppresses CC cells progression. **A** The mRNA expression of NR4A1 in CC were analyzed by GENT2 and GSE39001 datasets. For violin polts, midlines indicate the median, upper and lower lines indicate the first and third quartiles. **B** Representative images of IHC staining showed the protein levels of NR4A1 in CC tissues and paired adjacent nontumor tissues (n = 8, left) and quantitatively analyzed (right). **C** MTT assay showed the proliferative ability of CC cells. **D** The effect of NR4A1 on cell migration and invasion in CC cells. **E** The proliferative ability was investigated by colony formation. **F** Western blot assay of N-cadherin, Vimentin and E-cadherin protein levels in overexpression and knockdown of NR4A1 CC cells. All experiments were performed at least 3 independent times, and data are presented as means ± SD expect where otherwise specified. *P < 0.05, **P < 0.01, ***P < 0.001

Through analyzing the transcriptome data in GEPIA2, GEO (GSE7803, GSE7410 and GSE39001), GENT2 and TIMER2.0 databases, we found that NR4A1 mRNA expression was lower in CC tissues than in non-tumor tissues (Fig. 3A; Additional file 1: Fig.S3A–C). Similarly, our IHC assay also confirmed this change in protein levels (Fig. 3B). To further investigate the function of NR4A1 in CC, gain- and loss-of-function studies were performed (Additional file 1: Fig. S3D). Overexpression of NR4A1 significantly suppressed, while deletion of NR4A1 increased proliferation of CC cells by using MTT assay (Fig. 3C). The transwell migration and invasion assays revealed that NR4A1 upregulation significantly reduced migratory and invasive ability of CC cells. The opposite results were observed for NR4A1-silenced CC cell lines (Fig. 3D). In addition, colony formation assay indicated that overexpression of NR4A1 significantly suppressed cell proliferation in CC cells, while colony formation increased in shR-NR4A1 CC cells (Fig. 3E). Overexpression of NR4A1 resulted in downregulation of N-cadherin and Vimentin protein expression and promoted E-cadherin protein expression, whereas knockdown of NR4A1 caused the opposite effect (Fig. 3F).

To verify the effect of NR4A1 on METTL3-meditated cell progression in CC cells, we then performed a series of functional rescue experiments. As expected, overexpression of NR4A1 significantly abrogated METTL3-induced cell proliferation and clone formation in CC cells (Additional file 1: Fig.S3E and G). Indeed, forced expression of NR4A1 also impeded the migratory and invasive ability of CC cells in METTL3-overexpression background (Additional file 1: Fig.S3F). Taken together, all these data indicate that NR4A1 functions as tumor suppressor and dramatically abrogates the malignant phenotype induced by METTL3 in CC cells.

### YTHDF2 facilitates NR4A1 mRNA decay through m^6^A dependent pathway

**Fig. 4:**
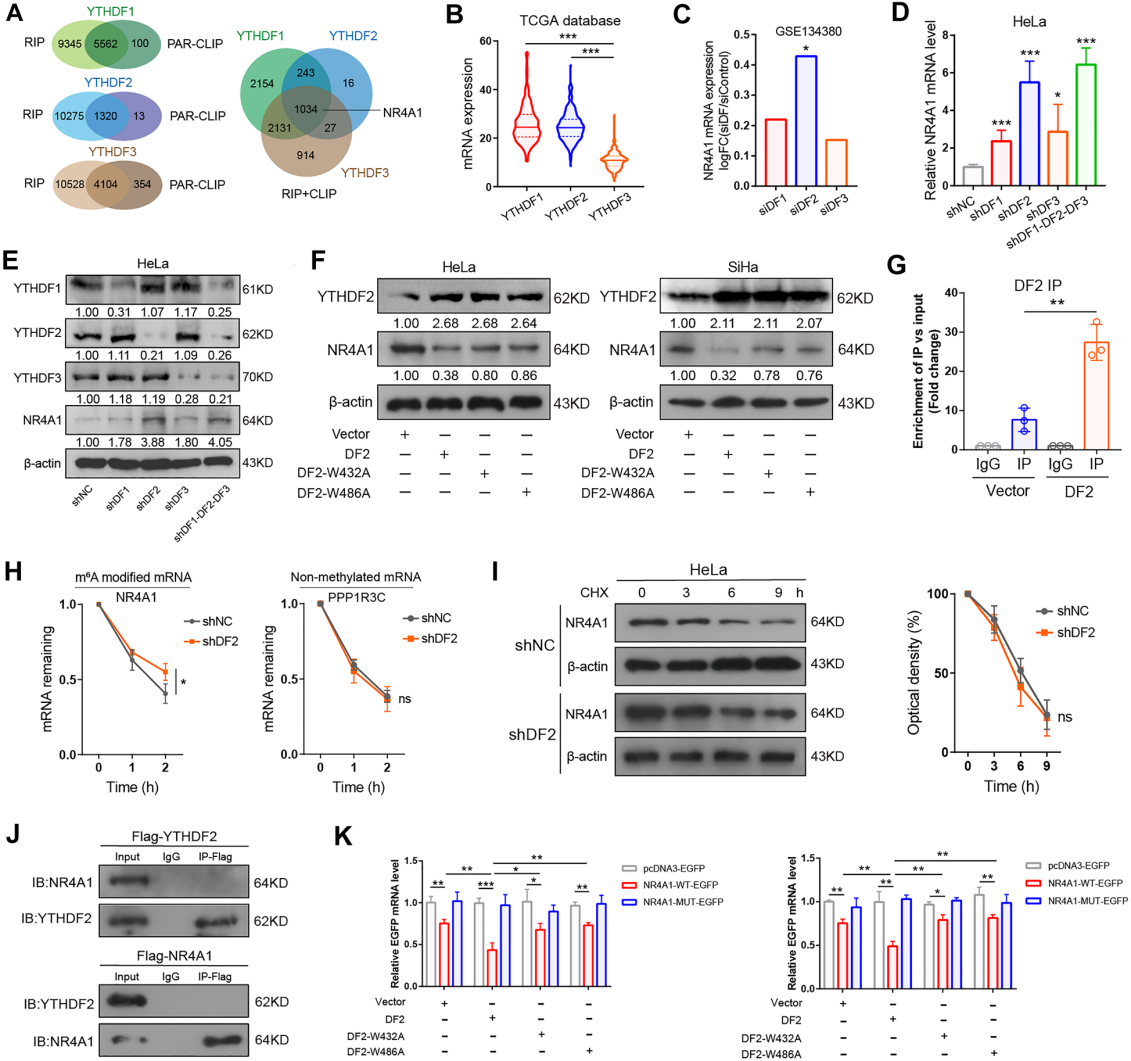
YTHDF2 sufficient to mediate the degradation of NR4A1 mRNA via m^6^A-dependent pathway. **A** Left: Overlap of YTHDF target genes identified by published RIP–seq and PAR-CLIP-seq in HeLa cells. Right: Venn diagram showed the candidate targets (PAR-CLIP + RIP targets) among all YTHDF paralogs. **B** The levels of YTHDF paralogs in TCGA-CC cohorts. Midlines indicate the median, upper and lines indicate the first and third quartiles. **C** The NR4A1 mRNA expression upon silencing YTHDF paralogs were analyzed by GSE134380 dataset. D-E, NR4A1 mRNA (**D**) and protein (**E**) levels were measured by RT-qPCR and western blot assay in HeLa cells. **F** Protein levels of NR4A1 in CC cells with overexpression wild-type (WT) or m^6^A recognition defective YTHDF2 (W432A and W486A) plasmids. **G** RIP-qPCR assay of the NR4A1 mRNA enrichment by YTHDF2 in overexpression YTHDF2 and control HeLa cells. **H** The stability of NR4A1 mRNA and non-methylated mRNA (PPP1R3C) upon the silencing of YTHDF2 were determined by RT-qPCR after treatment with act-D during indicated time points. **I** Left: The protein stability of NR4A1 was examined by western blot assay after treatment with CHX during indicated time points. Right: Quantification of protein stability. **J** The interaction between YTHDF2 protein and NR4A1 protein was measured via Co-IP assay. **K** The relative EGFP levels of co-transfected with the indicated plasmids, and normalized to the level of β-actin (left) and RFP (right) mRNA. All experiments were performed at least 3 independent times, and data are presented as means ± SD expect where otherwise specified. *P < 0.05, **P < 0.01, ***P < 0.001

It has found that specific members of the YTHDF family of m^6^A-binding proteins (YTHDF1, YTHDF2, and YTHDF3) control the fate of modified RNAs at specific m^6^A sites through different functions. In order to investigates the direct m^6^A reader proteins that mediate the repression of NR4A1 by m^6^A modification, we took advantage of available published RIP-seq and CLIP-seq datasets that identified RNA targets of YTHDF1, YTHDF2 and YTHDF3 in HeLa cells [6, 8, 9]. The 5562, 1320 and 4104 high-confidence transcripts identified by RIP data and PAR-CLIP data can be considered as candidate RNA targets of YTHDF1, YTHDF2 and YTHDF3, respectively (Fig. 4A). These three YTHDF proteins shared 1034 RNA targets, all of which include NR4A1 (Fig. 4A). TCGA database showed that the levels of YTHDF2 was similar to YTHDF1 (Fig. 4B). However, YTHDF2 was the most abundant YTHDF paralogs in CC cells (Additional file 1: Fig.S4A), which consistent with previous reports [11]. We then analyzed the RNA-seq of siR-YTHDF1, siR-YTHDF2 and siR-YTHDF3 in HeLa cells (GSE134380), with YTHDF2 having the most apparent impact on NR4A1 expression (Fig. 4C). Next, we identified the efficiency of overexpression and knockdown of YTHDF paralogs in CC cells (Fig. 4E, F, Additional file 1: Fig.S4C). Western blot and RT-qPCR assays were then performed to examine the levels of NR4A1 in silencing of the three YTHDF paralogs alone or together in HeLa cells. We observed all YTHDF paralogs act to affect the expression of NR4A1 (Fig. 4D, E). And the change of NR4A1 levels become evident when all three YTHDF paralogs were silenced simultaneously (Fig. 4D and E; Additional file 1: Fig.S4B). Importantly, among these three paralogs, the increase in NR4A1 mRNA levels and protein levels were most apparent when YTHDF2 was knocked down, implying that YTHDF2 might be a major reader for NR4A1 (Fig. 4D, E). Consistently, overexpression YTHDF2 significantly decreased the expression of NR4A1 in CC cells (Additional file 1: Fig.S4C, D). YTHDF2 relying on its hydrophobic amino acids W432 and W486 for specific recognition and binding of m^6^A-modified RNAs [28]. We therefore mutated the m^6^A recognition sites into W432A and W486A, which markedly decreased the binding affinity of YTHDF2 to m^6^A-containing RNA. In CC cells expressed of endogenous wild-type YTHDF2 (YTHDF2-WT), but not the catalytic inactive mutant YTHDF2 (YTHDF2-W432A and YTHDF2-W486A), decreased the levels of NR4A1 (Fig. 4F; Additional file 1: Fig. S4E). RIP-qPCR assay showed that NR4A1 mRNA was significantly enriched by YTHDF2 after abundantly expressed YTHDF2 (Fig. 4G). RT-qPCR assay indicated that knockdown of YTHDF2 increased the NR4A1 mRNA stability before 0 h, 1 h and 2 h after act-D treatment, but not non-methylated mRNA PPP1R3C [11] (Fig. 4H). Interestingly, increased NR4A1 mRNA stability was also evident upon triple knockdown simultaneously (Additional file 1: Fig. S4F). In addition, the change of NR4A1 protein stability was not apparent when YTHDF2 knockdown after incubation of CHX during indicated times (Fig. 4I). Co-IP analysis revealed that YTHDF2 protein was not directly interacted with NR4A1 protein (Fig. 4J). To ascertain whether YTHDF2 regulates the modification of NR4A1 mRNA through the same m^6^A site as METTL3, a series of experiments were carried out. Using western blot, RT-qPCR and fluorescence report assays, we found that exogenous YTHDF2 efficiently disrupted the activity of NR4A1-EGFP reporter in the presence of m^6^A motif, while overexpressed of YTHDF2-W432A-MUT and YTHDF2-W486A-MUT had no significant effect on EGFP expression of wild-type NR4A1-EGFP (Fig. 4K; Additional file 1: Fig. S4G, H). Taken together, our data demonstrate that YTHDF2 recognizes and binds the CDS region of NR4A1 to destabilize NR4A1 mRNA via an m^6^A-dependent mechanism.

### YTHDF2 destabilizes NR4A1 transcripts by DDX6

**Fig. 5:**
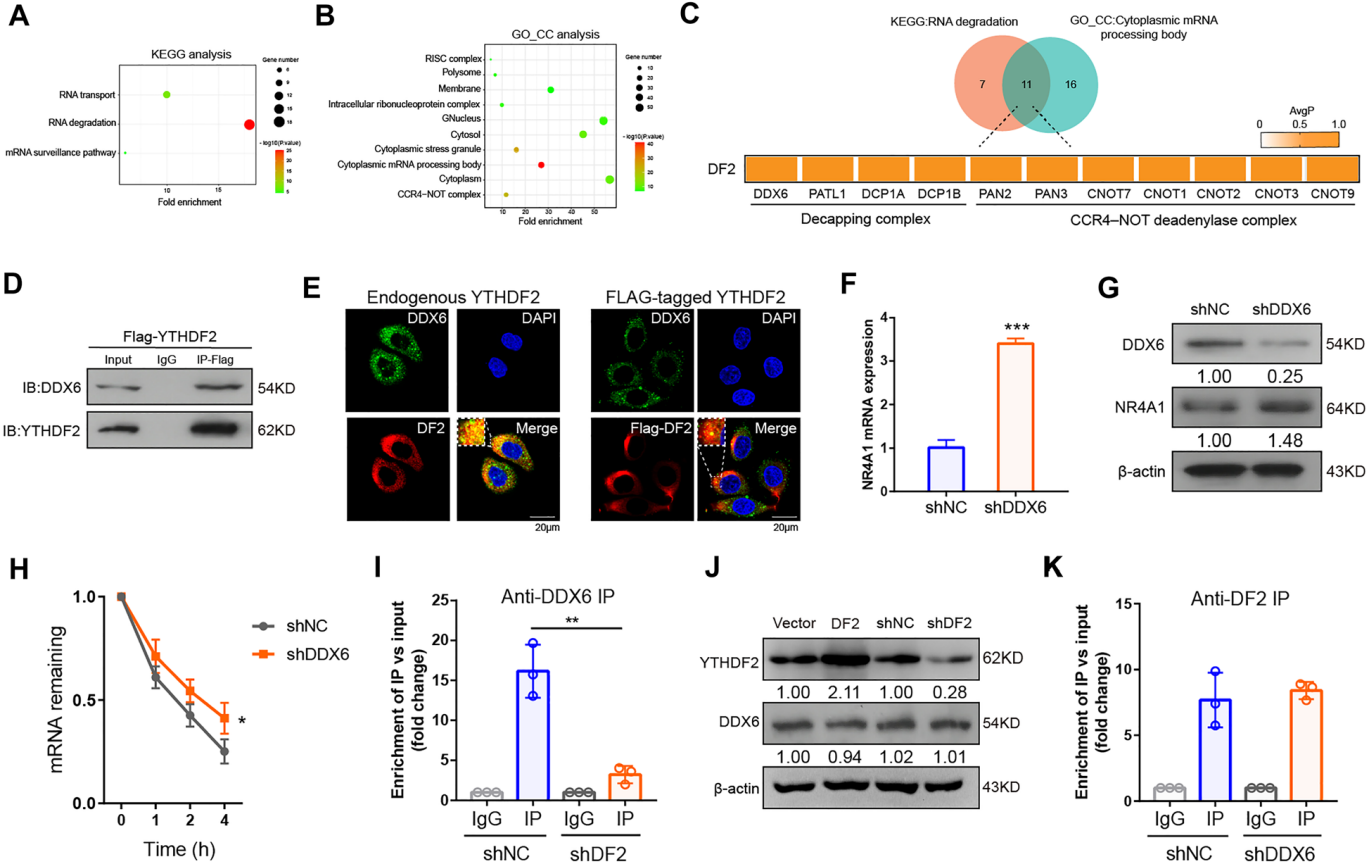
YTHDF2 recruits DDX6 to promote NR4A1 mRNA decay. **A**, **B** KEGG and GO pathway enrichment analysis of the high-confidence interacting proteins with YTHDF2. **C** Overlap of RNA degradation related genes and cytoplasmic mRNA processing body related genes as identified by **A** and **B**. **D** The interaction of YTHDF2 protein and DDX6 protein via Co-IP assay. **E** Confocal microscopy images indicated that YTHDF2 (red) were colocalized with P-bodies marker DDX6 (green). F-G, The NR4A1 mRNA (**F**) and protein (**G**) were determined by RT-qPCR and western blot assays upon silencing DDX6 in HeLa cells. **H** The mRNA stability of NR4A1 mRNA in HeLa cells after treatment with act-D during indicated time points in shR-DDX6 and shR-NC HeLa cells. **I** RIP-qPCR was used to determine the enrichment of NR4A1 in shR-NC and shR-YTHDF2 HeLa cells by using a DDX6-antibody. **J** The effect of YTHDF2 on DDX6 protein expression via western blot assay. K, RIP-qPCR was performed to measure the enrichment of NR4A1 in DDX6 knockdown and control cells using a YTHDF2-antibody. All experiments were performed at least 3 independent times, and data are presented as means ± SD. *P < 0.05, **P < 0.01, ***P < 0.001

To determine the molecular mechanism of how YTHDF2 to execute its mRNA degradation function to NR4A1, we analyzed a recent systematic in vivo proximity-dependent biotinylation (BioID) proteomic approach (http://prohits-web.lunenfeld.ca/) [29] of high-confidence interacting proteins for YTHDF2. The top 100 high-confidence interactors of YTHDF2 were selected, which are mainly involved in RNA degradation and cytoplasmic mRNA processing body (P- body) and so on (Fig. 5A, B). Through overlap analysis, we identified 11 high-confidence candidate targets. Notably, these 11 high-confidence interactors included the RNA helicase DDX6, a key component of eukaryotic P-body assembly and mRNA decapping complex [30, 31] (Fig. 5C). In addition, Co-IP assay also indicated that DDX6 was an interactor of YTHDF2 (Fig. 5D). We also visualized that YTHDF2 was colocalized with DDX6 using confocal fluorescence microscopy (Fig. 5E). Then we sought to determine whether DDX6 affects the levels of NR4A1. Our data showed that the mRNA and protein levels of NR4A1 were upregulated in DDX6 knocked down HeLa cells (Fig. 5F, G). Consistently, the stability of NR4A1 mRNA also increased upon depletion of DDX6 (Fig. 5H). To determine whether YTHDF2 binding is required for DDX6-mediated degradation of NR4A1 mRNA, RIP-qPCR experiments were performed. DDX6 enrichment of NR4A1 mRNA was decreased in YTHDF2 depleted cells (Fig. 5I). In addition, overexpression and knockdown of YTHDF2 had no effect on DDX6 protein levels (Fig. 5J) Nevertheless, YTHDF2 still able to bind NR4A1 transcript upon DDX6-silenced HeLa cells (Fig. 5K). Collectively, these data suggest that YTHDF2 mediates NR4A1 expression through a DDX6-dependent RNA degradation mechanism.

### Upregulation of YTHDF2 induces malignant phenotype of CC cells, which can be mitigated by NR4A1

**Fig. 6:**
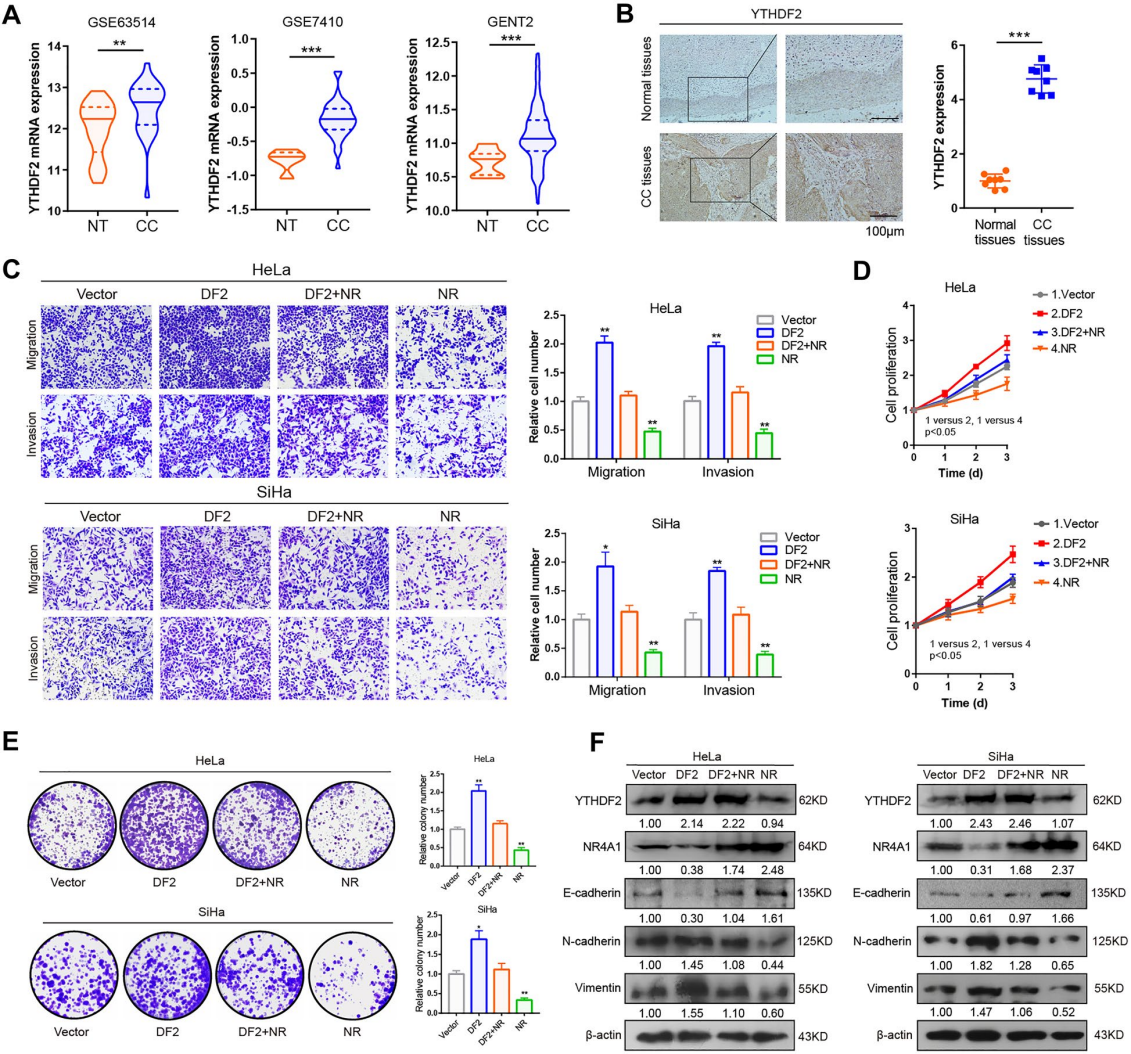
NR4A1 attenuates malignant progression that induced by YTHDF2 in CC cells. **A** GEO database (GSE7410 and GSE63514) and GENT2 database of YTHDF2 mRNA in CC cohorts. The three lines inside the violin plots indicate the first quartile, median and third quartile. **B** Representative images of IHC staining for YTHDF2 protein from CC tissues and matched adjacent non-tumor tissues (n = 8, left) and quantitatively analyzed (right). **C** Transwell migration and invasion assays in CC cells expressing the specified plasmids. **D**, **E** The MTT (D) and colony formation assays (**E**) showed the cell proliferative ability with transfected the indicated plasmids in CC cells. **F** Western blot assay showed that the expression of N-cadherin, Vimentin and E- cadherin with transfected the indicated plasmids in CC cells. All experiments were performed at least 3 independent times, and data are presented as means ± SD except where otherwise specified. *P < 0.05, **P < 0.01, ***P < 0.001

We next explored the function of YTHDF2 in CC progression. The mRNA expression of YTHDF2 was evidently elevated according to GEO (GSE7410, GSE63514), GENT2 and TIMER2.0 databases (Fig. 6A; Additional file 1: Fig. S5). Consistently, IHC analysis revealed that the protein levels of YTHDF2 were also increased compared to the non-tumor controls (Fig. 6B). Next, to validate whether YTHDF2 affect for CC progression, the gain- and loss-of-function studies were used. The transwell migration and invasion results indicated that migratory and invasive capacities were obviously boosted in YTHDF2 overexpressed CC cells, which are blocked by co-expression of NR4A1 (Fig. 6C). In addition, overexpression of YTHDF2 significantly enhanced the cell proliferation and clone formation in CC cells. However, when NR4A1 was reintroduced into the cells, the aggressive cancer progression promoted by overexpressed YTHDF2 was substantially hindered (Fig. 6D, E). Additionally, forced expression of YTHDF2 apparently increased the protein levels of N-cadherin and Vimentin as well as decreased the protein levels of E-cadherin, while these effects were significantly impaired when NR4A1 was reintroduced (Fig. 6F). Collectively, our results suggest that NR4A1 antagonizes YTHDF2-mediated malignant phenotype of CC cells.

### YTHDF2-NR4A1 axis regulates the transcription repression of AKT1 by recruiting LSD1/HDAC1/CoREST complex

**Fig. 7:**
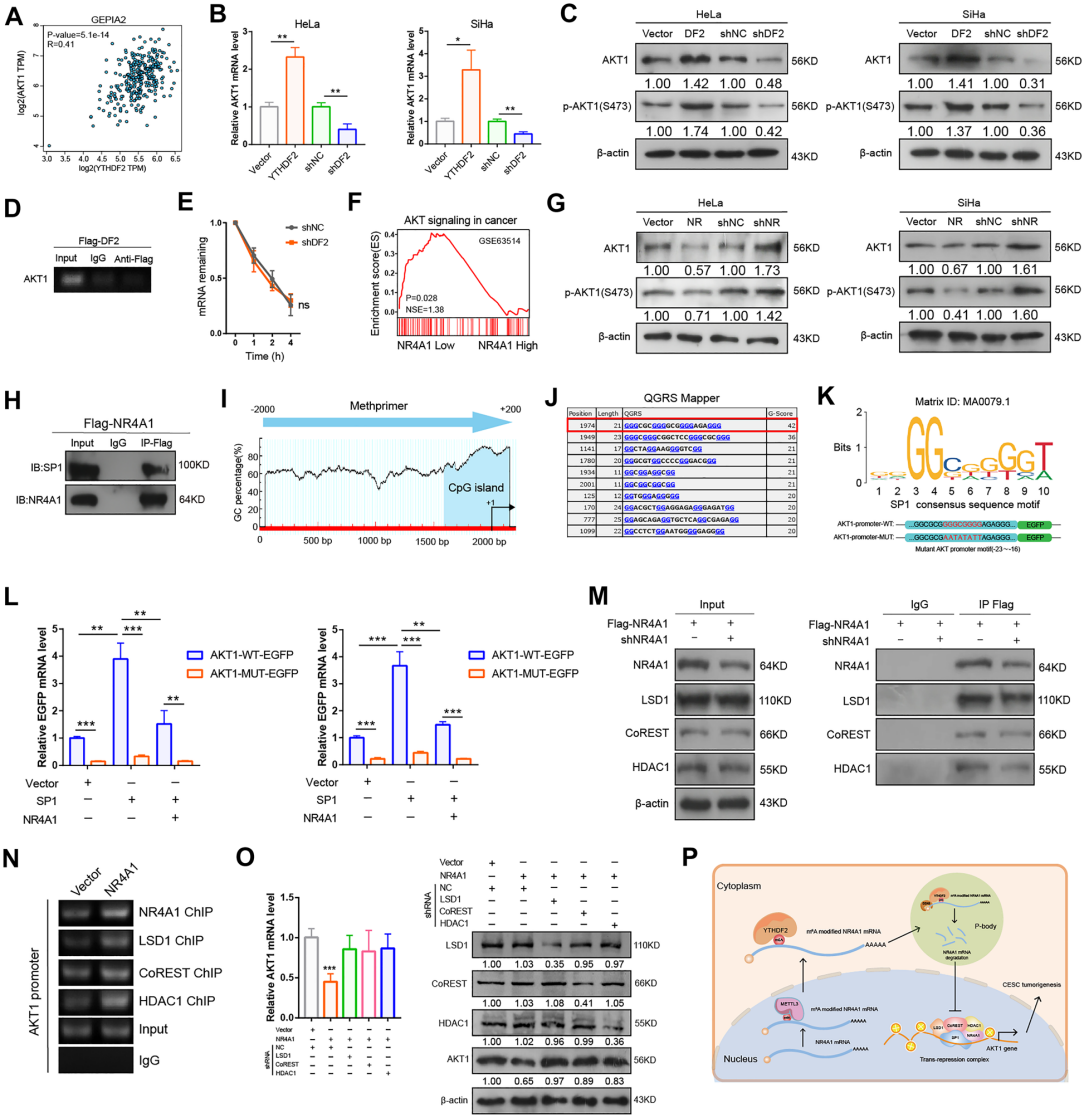
YTHDF2-NR4A1 axis meditates the transcription repression of AKT1 by recruiting LHC complex. **A** YTHDF2 was positively correlated with AKT1 in GEPIA2 database. **B** The mRNA levels of AKT1 were detected by RT-qPCR with overexpression and knockdown of YTHDF2. **C** Western blot assay was used to detect the alteration of AKT and AKT phosphorylation after overexpression and knockdown of YTHDF2 in CC cells. **D** RIP assay of the enrichment of AKT1 transcripts by YTHDF2. **E** RNA remaining for AKT1 in HeLa cells transfected with control and YTHDF2 knockdown were determined by RT-qPCR after treated with act-D at indicated times. **F** GSEA identified a significant association between NR4A1 and AKT pathway. **G** Western blot showed the protein levels of AKT1 and p-AKT1 after overexpression and knockdown of NR4A1 in CC cells. **H** Co-IP assay was performed to analyze the interaction between NR4A1 protein and SP1 protein. **I**–**K** The analysis of SP1-bound site of AKT1 promoter. **L** The EGFP reporter assay was preformed to measure the EGFP levels by co-transfected with the indicated plasmids. β-actin (left) and RFP (right) were used for normalization. **M** Binding of NR4A1 to LSD1, CoREST and HDAC1 in HeLa cells as shown by Co-IP assay. **N** ChIP assay of AKT1 promoter binds of NR4A1 and components of LHC complex. **O** The mRNA and protein expression were measured by RT-qPCR (left) and western blot assay (right) after transfected of shR-LSD1, shR-CoREST and shR-HDAC1, respectively, upon a NR4A1-overexpression background. **P** All findings in this study are presented as a schematic diagram. All experiments were performed at least 3 independent times, and data are presented as means ± SD. *P < 0.05, **P < 0.01, ***P < 0.001

We next to investigate the underlying mechanisms of YTHDF2-NR4A1 axis in regulation of tumorigenic potential in CC cells. Recent studies indicated that YTHDF2 regulated AKT pathway in cancers [15, 32]. Consistent with previous report, we found that the expression of YTHDF2 was evidently positively corrected with AKT1 expression in cervical cancer using GEPIA2 database (Fig. 7A). In addition, our results confirmed that the levels of AKT1 and p-AKT1(S473) were evidently increased by overexpression of YTHDF2 (Fig. 7B, C). However, knockdown of YTHDF2 caused an opposite result in CC cells (Fig. 7B, C). It is worth noting that the transcript of AKT1 is modified by m^6^A according to previous report [11]. Therefore, we sought to determine whether YTHDF2 mediates the levels of AKT1 through m^6^A-dependent manner. A previous PAR-CLIP-seq showed that AKT1 was not a high-confident target by YTHDF2 [11]. Then, our RIP-PCR results also indicated that YTHDF2 could not enrich the predicted highest-confidence m^6^A site in AKT1 transcripts based on SRAMP (a sequence-based m^6^A modification site predictor) [33] (Fig. 7D; Additional file 1: Fig. S6A). Furthermore, the stability of AKT1 mRNA was also unaffected by YTHDF2 silencing (Fig. 7E), suggesting that AKT1 is unlikely to be a direct target mRNA by YTHDF2-dependent m^6^A regulatory mechanism. Next, we hypothesized that YTHDF2 activates the AKT pathway by inhibiting NR4A1. GSEA was performed using GSE39001 of CC data for this hypothesis. We found that NR4A1 was correlated with AKT pathway in cancer (Fig. 7F). Previous studies have shown that AKT1 is extensively involved in these biological processes. As expected, knockdown or overexpression of NR4A1, respectively, activated or inactivated the AKT pathway in CC cells, implying that there must exist a special molecular mechanism controlling AKT1 activity by NR4A1 (Fig. 7G). NR4A1 is an orphan nuclear receptor that functions as a transcription factor in multiple cancers. However, we did not find any DNA response elements in the AKT1 promoter that bound to NR4A1 through silico analysis. According to previous study, NR4A1 has been shown to recruit LSD1/CoREST/HDACs complex and interact with SP1 to regulate the transcription of candidate genes [34]. To prove this hypothesis, we first identified that NR4A1 was an interactor with SP1 using Co-IP analysis (Fig. 7H). To our surprise, AKT1 promoter contains many G-rich regulatory elements including G4 structures (Fig. 7I, J), which have been reported to participate in regulating the transcription of many target genes by being recognized by SP1 [35, 36]. We therefore analyze the G-rich regions by Methprimer program [37], we found the CpG island was near the transcription start site (TSS) of AKT1 promoter (Fig. 7I). Next, we predicted potential SP1 binding sites in AKT1 promoter by QGRS Mapper (G-quadruplexes prediction algorithm) [38] and JASPAR [39] (Fig. 7J, K). One segment (nucleotide sequence from − 35 to -15, containing potential motifs of SP1: GGGGCGGGGA) near the transcription start site (TSS) of AKT1 promoter was selected for functional analysis (Fig. 7K). SP1 significantly advanced the EGFP activity of a AKT1-EGFP reporter containing wild-type promoter sequences, which could be alleviated by co-expression of NR4A1. Nevertheless, the EGFP activity of AKT1 promoter containing mutated SP1 binding site was only slightly responsive to overexpression of SP1 or NR4A1(Fig. 7L; Additional file 1:Fig. S6C, D). To ascertain whether NR4A1 was associated with LSD1/HDAC1/CoREST (LHC) complex. We performed Co-IP experiments in HeLa cells. As expected, NR4A1 was an interactor with LSD1, HDAC1 and CoREST (Fig. 7M). Notably, NR4A1 had no affected on the expression of all components of LHC complex (Additional file 1: Fig. S6B). Then we designed a primer set covering SP1-binding site and performed the ChIP assay in overexpressed-NR4A1 and control HeLa cells. The results demonstrated that NR4A1 induced binding of LHC complex to SP1 binding site in AKT1 promoter (Fig. 7N). Finally, the attenuation of AKT1 caused by NR4A1 was alleviated when knockdown of LSD1, CoREST and HDAC1, respectively (Fig. 7O). In summary, these findings reveal that YTHDF2-NR4A1 axis regulates the AKT pathway by recruiting LHC complex to inhibit the transcription of AKT1.

## Discussion

m^6^A is the most abundant and prevalent chemical modification of adenosine in RNA that plays wide-ranging roles in the regulation of RNA processes [2]. Accumulating evidence suggests that m^6^A modification modulates all stages in the life cycle of RNA, including mRNA splicing, nuclear export, stability and translation. METTL3, acting as the core component of N^6^-methytransferse complex, has been reported to be involved in a variety of tumor biological progression, including cervical cancer [1, 2, 12, 14]. Previously, Du et al. suggested that the high expression of METTL3 in cervical cancer promoted tumor progression through regulating of TXNDC5 expression in an m^6^A-dependent mechanism [17]. Similarly, another study indicated that METTL3-mediated m^6^A modification on HK2 could accelerate the Warburg effects in cervical cancer [18]. In the present study, we revealed that the expression of METTL3 was significantly increased in CC tissues and cells, and obviously promoting the malignant phenotype of CC cells. To further elucidate the molecular mechanism of METTL3, we combined the data from experimental and in silico analysis, revealing that NR4A1 was the target mRNA of METTL3. Mechanistically, our data showed that METTL3 regulated the mRNA stability of NR4A1 through m^6^A-dependent manner.

It has been reported that the m^6^A reader protein directly recognizes and binds m^6^A motifs to affect the fate of target mRNAs. Notably, recent study suggested that YTHDF family proteins functional similar to trigger degradation of m^6^A-mRNAs in HeLa cells [11]. To identify the role of YTHDF paralogs in regulation of NR4A1 transcript, we analyzed the published RIP-seq and CLIP-seq and found that NR4A1 might be the downstream target of all YTHDF paralogs. Furthermore, our in vitro data confirmed that knockdown of any of YTHDF protein was involved in the repression of NR4A1 mRNA, and YTHDF2 was the most effective regulator. Although YTHDF proteins appeared to exhibit similar roles in contributing NR4A1 mRNA degradation, silencing of different YTHDF paralogs has different effects. This may be due to that YTHDF2 is more abundant than other YTHDF proteins in HeLa cells [7, 11]. Meanwhile, the cellular context, protein interaction networks, tissue or cell specificity and the localization also affect the function of YTHDF proteins. Here, we also found depletion of YTHDF2, not YTHDF1 or YTHDF3, increased NR4A1 mRNA stability, while overexpression of NR4A1 attenuated YTHDF2-induced malignant progression of CC cells. Therefore, multiple lines of evidence sustained our notion that YTHDF2 serve a dominant function in regulation of NR4A1 expression via m^6^A-dependent mechanism in CC context. YTHDF2 consists of a C-terminal YTH domain and a N-terminal P/Q/N-rich domain, the former as an essential component to facilitate m^6^A-containing RNA binding and the latter as the subunit for recruiting the mRNA to cytoplasmic foci (such as P-body) to support mRNA degradation [8]. In general, mature eukaryotic mRNAs are protected by a 5’ cap structure and a 3’ poly-A tail. As soon as the mRNAs reach cytosol, the exonuclease begins to control the length of the poly-A tail. Once receiving a specific degradation signal, two general mechanisms exist for effectively destroying the target mRNA: the 5’-to-3’ degradation that start with the removal of 5’ cap (also called decapping) and the “exposed” mRNA is rapidly decayed, the 3’-to-5’ degradation that the mRNA continues to be degrade from its 3’ poly-A tail (also called deadenylation). Moreover, these two processes can occur in parallel on the same mRNA molecular [40, 41]. According to a previous study, YTHDF2 recruited the CCR4–NOT deadenylase complex to cause deadenylation and degradation of the transcripts, partially through a directly interaction between the N terminus of YTHDF2 and the SH domain of the CCR4-NOT transcription complex subunit 1 (CNOT1) [7]. In addition to the aforementioned deadenylation pathway, we speculate that whether exist a 5’-to-3’ degradation manner to destroy its target mRNA. To test this hypothesize, we firstly reanalyzed a recent Bio-ID study [29] and found that decapping RNA degradation complexes were among the high-confidence interactors of YTHDF2 in these experiments. Then we discovered that YTHDF2 regulated the expression and stability of NR4A1 mRNA through a DDX6-dependent RNA degradation manner. P-body is a cytosolic membraneless ribonucleoprotein particle (RNP) granules exist throughout the cell and plays a role in mRNA degradation. This structure is assembled by translationally inactive mRNA and different mRNA-binding proteins [42]. Among them, the RNA helicase DDX6 is a key protein for human P-body assembly [30]. Various studies indicated that DDX6 localized in P-body and associated with mRNA decapping complex, which serves as a repression and decay cofactor to support mRNA degradation for contributing RNA metabolism [31, 43]. Previous study indicated that DDX6 prevent premature differentiation of progenitor cells through destroying 5’-cap of KLF4 mRNA [31]. Overall, our data provided a new insight into the function of YTHDF2-modified RNA degradation mechanism via recruiting decapping complex component DDX6.

NR4A1 is an orphan nuclear receptor containing an N-terminal transactivation domain (TAD), a conserved central double zinc finger DNA-binding domain (DBD) and a ligand-binding domain (LBD) at C-terminal, and its nuclear localization signal (NLS) is located in the DBD domain [44, 45]. NR4A1 has been linked to a wide range of cancers. Depending on the subcellular localization, intracellular abundance and transcriptional activity, NR4A1 functions either as a tumor suppressor or enhancer in many cancer types [26]. Usually, the high level of endogenous NR4A1 exhibits a protumor function through multiple mechanism pathways in various tumors [46]. However, several studies also indicated that NR4A1 has proapoptotic and tumor inhibitory effects [47–50]. Importantly, a recent study revealed that NR4A1 implicated in the anti-tumor effect to induce cell apoptosis in cervical cancer [25]. This is consistent with our results, and implies that NR4A1 acts as a tumor suppressor in modulating the malignant phenotype of cervical cancer cells. Mechanistically, our study showed that NR4A1 was responsible for binding SP1 on the AKT1 promoter region and recruiting the LHC transcriptional repression complex to inhibit AKT1 transcriptional activity.

The LHC complex is unique in that it removes both histone methyl and acetyl modifications through its active components of LSD1 and HDAC1. In the context of LHC complex, LSD1 specifically removes methyl groups from H3K4me1/2, and HDAC1 seems to preferentially deacetylate H3K9ac and H3K14ac in nucleosome substrates [51, 52]. Indeed, we noticed that there are some reports about the protumor role of LSD1 and HDAC1 in CC progression [53–55]. However, a previous work revealed that HDAC1 is associated with the suppression of OCT4 in cervical cancer cells [56]. OCT4 is a key transcription factor and supports biology process of cervical cancer [57]. Meanwhile, Hung-Cheng Lai group found that TET1 interacts with LSD1 to form a transcriptional repression complex on the ZEB1 and VIM promoter regions to inhibit malignant phenotypes in cervical precancerous cells, thereby suppressing early carcinogenesis [58]. It seems that LSD1 and HDAC1 function either as a tumor suppressor or enhancer, depending on the cellular context and tumor stage. As we know, the effect of entire LHC components have not been detected in CC context previously because these proteins are typically depleted or overexpressed separately rather than simultaneously. We considered that LHC complex may act redundantly to control epigenetic reprogramming depending on various extracellular and intracellular stimuli in different cellular context, and depletion of only one protein may allow for varying degrees of compensation by the other LHC components. However, the exact function of a particular protein and entire LHC complex components in CC and other cancer need further investigation.

A previous study has shown that SP1 is a positive regulator that induces transcriptional activation of AKT1 [35]. Moreover, our results further suggested that this activity of SP1 was repressed by overexpression of NR4A1. We then considered the possibility that LHC complex may be functionally redundant and that it also supports deacetylation and demethylation of SP1, thereby inhibiting the transcription activity of SP1 [59, 60]. Additionally, we noticed that overexpression of NR4A1 was associated with an increase of LHC components on the specific region of endogenous AKT1 promoter, but not at the overall level. These results imply that the LHC complex may be responded to the recruitment signal of NR4A1 and thereby alters its nuclear localization, but does not affect its overall abundance in cells. AKT1 mediates various aspects of cancer development and progression, including in CC cells. Once activated, the AKT1 pathway reprograms cellular metabolism, thereby inducing cell growth, proliferation and survival in cancer cells [61]. Overall, these data support the idea that the overexpression of NR4A1 induces the transcriptional repression of AKT1 through recruiting LHC complex. Therefore, low expression of endogenous NR4A1 cannot efficiently manipulate transcriptional repression of AKT1 in CC context.

One of the limitations of this study is that only a small number of clinical samples from CC patients were used. Moreover, as a previous study revealed that METTL3 relocates in cytoplasm and exerts an m^6^A-independent function to regulate cancer progression [62], further researches are needed to investigate the functional complexity of METTL3 both in cytoplasm and nucleus. Finally, with accumulating evidence for the functional diversity of m^6^A modification during cancer processes, exploring the therapeutic potential of targeting m^6^A regulators and their target RNAs may be beneficial for tumor therapy and provide novel approaches for designing more effective anti-tumor treatments.

## Conclusions

In summary, we provided compelling evidence indicating that YTHDF2 is responsible for degradation of NR4A1 mRNA via binding and recognizing the m^6^A modification by METTL3, and therefore alleviating NR4A1-induced transcription repression in AKT1 by recruiting LHC complex. Since a great number of m^6^A regulators are expressed and involved in manipulating the fate of cells, we cannot exclude the possibility that m^6^A modification regulators modulate other targets to control the cell progression. Taken together, our study provides a novel regulatory mechanism to explain the malignant characteristics of CC cells, and may expand our understanding to develop therapeutic strategies for CC.

## Methods

### Specimens, cell culture and transfection

All clinical specimens were collected from Tangshan People’s Hospital and histopathologically confirmed by the pathologists. Detailed information about the specimens is listed in Additional file 3: Table S3. Written informed consent was obtained from all patients. This study was approved by the Ethics Committee of Tangshan People’s Hospital. Human cervical cell line HeLa, SiHa, C-33A and CaSki were purchased from the American Type Culture Collection (ATCC, Manassas, VA) and cultured in Dulbecco’s modified Eagle medium (DMEM, GIBCO) with 10% fetal bovine serum (FBS, GIBCO) and 1% PS (100 units/ml penicillin, 100 ug/ml streptomycin), and incubated at 37 °C in a humidified atmosphere with 5% CO_2_. Immortalized human cervical epithelial cell line S12 was kindly provided by Prof. Wang (Huazhong University of Science and Technology). Cell transfection was performed with Lipofectamine 2000™ reagent (Invitrogen) according to the manufacturer’s protocol.

### RNA extraction and reverse transcription-quantitative PCR (RT-qPCR) for gene expression

Total RNA was extracted from cells using TRIzol regent (Invitrogen, USA) according to the manufacturer’s protocol. Then 500 ng RNA were reversely transcribed into cDNA with HiScript II 1st Strand cDNA Synthesis Kit (Vazyme). The RT-qPCR was performed on 7500 Fast Real-Time PCR system with 2 × Universal SYBR GreenFast qPCR Mix (ABclonal) to determine target RNA levels. All results were normalized to the mRNA levels of β-actin. The primers used in RT-qPCR were listed in Additional file 4: Table S1.

### mRNA stability

To measure the mRNA stability, 5 µg/ml actinomycin D (act-D, Genview) was treated to cells for the indicated times. Then, cells were collected and RNA was extracted by TRIzol. RNA was measured by RT-qPCR. All results were normalized to β-actin.

### RIP-qPCR

RIP assays were performed using the Magna RIP™ Kit (Millipore) according to the manufacturer’s recommendations. Briefly, RIP lysis was used to lyse cells for 5 minutes at 4 °C. Cell lysate was divided into three aliquots: input, experiment, and negative control.

The input sample was stored at − 80 ^o^C for later experiments. Two additional sets of RIP lysate were incubated with indicated antibody-conjugated beads overnight at 4  °C. After washing and purification, qPCR was performed to measure the target RNA expression. The primers used in RIP-qPCR were listed in Additional file 2: Table S1.

### EGFP reporter assay

To evaluate the m^6^A modification site of CDS in NR4A1, NR4A1-WT-EGFP, NR4A1-MUT-EGFP and control vector were co-transfected with pcDNA3.1-METTL3, pcDNA3.1-METTL3-MUT, pcDNA3.1-YTHDF2, pcDNA3.1-YTHDF2-MUT(W432A) or pcDNA3.1-YTHDF2-MUT(W486A) in 24-well plate, respectively. To evaluate the promoter activity of AKT1, AKT1-WT-EGFP and AKT1-MUT-EGFP were co-transfected with pcDNA3.1-SP1 and/or pcDNA3.1-NR4A1 in 24-well plate, respectively. The RFP expression vector was included for transfection normalization. After transfection for 48 h, the EGFP activity were measured with a fluorescence microscopy, western blot and RT-qPCR assays.

### Western blot assay

Western blot analysis was performed as previously described [63]. The primary antibodies used in this study were anti-β-actin (immunoway), anti-NR4A1 (immunoway), anti-METTL3 (abcam), anti-YTHDF1(Saier Biotechnology), anti-YTHDF2 (abcam), anti-YTHDF3(Saier Biotechnology), anti-AKT1 (Saier Biotechnology), anti-p-AKT1(S473) (Wanlei Biotechnology), anti-DDX6 (Saier Biotechnology), anti-LSD1 (Saier Biotechnology), anti-CoREST (Saier Biotechnology), anti-HDAC1 (Saier Biotechnology), anti-EGFP (Saier Biotechnology), anti-Vimentin (abcam), anti-N-Cadherin (abcam), anti-SP1 (abcam), anti-E- Cadherin (abcam) and anti-Flag (MBL). β-actin was the internal reference.

### Protein stability

To measure the mRNA stability, shRNAs were used to knockdown specific expression for 2 days. Then, cells were incubated with cycloheximide (CHX, Genview) at 100 µg/ml during indicated times. The protein levels were examined by Western blot analysis.

### Co-Immunoprecipitation (Co-IP) assay

Co-Immunoprecipitation (Co-IP) assay were performed as previously described with minor modifications [64]. Briefly, cells were collected and lysed in the lysis buffer containing 5 mM MgCl_2_, 20 mM imidazole, 300 mM KCl, 1% TritonX-100, 5% glycerol and 1× protease inhibitor cocktail (Beyotime) at 4 °C for 30 min. And then incubated with in dictated antibody-conjugated beads overnight at 4 °C. After incubation and washing, the immunoprecipitates were incubated with 3 × FLAG peptide (Beyotime) at 4 °C for 2 h. Then the eluates were immunoblotted with specified antibodies.

### Animal studies

All animal experiments were approved by Tianjin Medical University Animal Care and Use Committee. Six-week-old female BALB/c nude mice were used. For subcutaneous xenograft mouse models, a total of 5 × 10^6^ stably transduced (shR-NC and shR-METTL3) HeLa cells were subcutaneously injected into the nude mice to examine tumor growth. Six mice were included in each group. Tumor volume was measured every 2 days and calculated using the formula V = length diameter × (width diameter)^2^ × 1/2. The mice were killed at 15 days after injection, and tumors were removed and weighed for further studies. For in vivo lung metastasis model, 2 × 10^6^ stably transduced shR-NC and shR-METTL3 HeLa cells were injected into tail vein of nude mice (n = 4 for each group). After 2 months of injection, mice were sacrificed and metastatic lung tumors were analyzed.

### Statistical analyses

All analyses were conducted using GraphPad Prism 7 for Windows (GraphPad Software Inc., USA). All the experiments were performed at least three independent biological replicates, and the results were shown as means ± standard deviation (SD) except where otherwise specified. Student’s two-tailed t-test was used for statistical. P values < 0.05 were considered significant (*P < 0.05, **P < 0.01, ***P < 0.001, ns, no significant).

## Supplementary Information

**Additional file 1. **Supplementary methods and figures. **Fig. S1.** METTL3 exerts aprotumor function in CC. **Fig. S2.** METTL3 facilitates NR4A1 mRNA decaythrough m^6^A modification. **Fig. S3.** NR4A1 inhibitsMETTL3-induced CC cells progression. **Fig. S4. **YTHDF2 promotesNR4A1 decay through m^6^A-dependent mechanism. **Fig. S5 **TIMER2.0database of YTHDF2 mRNA in CC tissues. **Fig. S6.** YTHDF2-NR4A1 axis promotestranscriptional repression of AKT1 in CC cells**. **

**Additional file 2. Table S2. **Direct RNA-seq.

**Additional file 3. Table S3. **Characteristics of specimens.

**Additional file 4. Table S1. **The primers used in this study.

## Data Availability

All data created and analyzed during this work are involved in this published article and its supplementary information files.

## Abbreviations

CC: Cervical cancer
m^6^A: N^6^-methyladnosine
METTL3: Methyltransferase 3
YTHDF1/2/3: YTH domain–containing family protein 1/2/3
METTL14: Methyltransferase 14
WTAP: WT1 associated protein
FTO: FTO alpha-ketoglutarate dependent dioxygenase
ALKBH5: AlkB homolog 5
NR4A1: Nuclear receptor subfamily 4 group A member 1
AKT1: AKT serine/threonine kinase 1
DDX6: DEAD-box helicase 6
SP1: Sp1 transcription factor
LSD1: Lysine demethylase 1A
HDAC1: Histone deacetylase 1
CoREST: REST corepressor 1
IHC: Immunohistochemistry
RT-qPCR: Reverse transcription-quantitative PCR
Co-IP: Co-Immunoprecipitation
RIP: RNA immunoprecipitation
ChIP: Chromatin immunoprecipitation
