# Supplementary material for: RNA N6-methyladenosine modification mediates downregulation of NR4A1 to facilitate malignancy of cervical cancer
Source: Cell Biosci. 2022 Dec 25;12:207. doi: 10.1186/s13578-022-00937-w (PMC9790124; doi:10.1186/s13578-022-00937-w)
Supplement: Supplementary file 1 — Additional file 1. Supplementary methods and figures. Fig. S1. METTL3 exerts aprotumor function in CC. Fig. S2. METTL3 facilitates NR4A1 mRNA decaythrough m6A modification. Fig. S3. NR4A1 inhibitsMETTL3-induced CC cells progression. Fig. S4. YTHDF2 promotesNR4A1 decay through m6A-dependent mechanism. Fig. S5 TIMER2.0database of YTHDF2 mRNA in CC tissues. Fig. S6. YTHDF2-NR4A1 axis promotestranscriptional repression of AKT1 in CC cells. [file 13578_2022_937_MOESM1_ESM.docx]

**Supplementary information for**

**RNA N^6^-methyladenosine modification mediates downregulation of NR4A1 to facilitate malignancy of cervical cancer**

Tao Yu^1, †,^ *, Fuxia Wu^1, †^, Yan Jia^1, †^, Xue Zhang^1^, Xiaozhen Qi^1^, Zeyuan Jin^1^, Tongxin Hao^1^, Jianing Zhao^1^, Ziyu Liu^1^, Chaokun Wang^3^, Minmin Niu^1^, Qin Yue^1^, Min Li^1^ and Yankun Liu^2,^*

^1^ Department of Pathogen Biology, School of Basic Medical Sciences, Tianjin Medical University, Tianjin 300070, China

^2^ Department of Molecular Diagnosis, Tangshan People’s Hospital, Tangshan 063001, China;

^3^ Department of Integrative Chinese and Western Medicine, School of Basic Medical Sciences, Tianjin Medical University, Tianjin 300070, China

**Correspondence**: Tao Yu (ytao2020@tmu.edu.cn; ytao126126@126.com); Yankun Liu (rmyy_lyk@163.com)

These authors contributed equally: Tao Yu, Fuxia Wu and Yan Jia

**This PDF file includes:**

Supplementary Methods

Supplementary Figures 1-6

**Supplementary Methods**

**Plasmid, shRNA and generation of stable cell lines**

Human CDS of NR4A1, METTL3, and YTHDF2 were cloned into pcDNA3.1 with FLAG tag at N-terminus to generate over expression plasmid. Human CDS of SP1 was cloned into pcDNA3.1 with HA tag at N-terminus to generate SP1 expression plasmid. pcDNA3.1 was used as the control for analysis. METTL3 mutant (D395A/W398A) and YTHDF2 mutant (W432A and W486A) plasmids were constructed from previous studies [1,2]. For NR4A1, METTL3, YTHDF1, YTHDF2, YTHDF3, DDX6, LSD1, HDAC1 and CoREST knockdown, we constructed the shRNA plasmid by annealing double-strand hairpin cDNA and cloning the annealed duplex RNAi oligonucleotides into the pSilencer 2.1-U6 neo vector. The CDS fragments of NR4A1 containing m^6^A modification site and containing mutant m^6^A modification site were obtained by annealing double-strand DNA and inserting it into the pcDNA3.1-EGFP vector. The AKT promoter region was inserted into pGL3-EGFP vector, which generated wild type (WT) reporter. The mutant AKT1-promoter fragment was obtained by PCR-based site-directed mutagenesis and inserted into pGL3-EGFP vector to generate mutant reporter. To generate knockdown of METTL3 stable cells, HeLa cells were transduced with lentiviruses expressing METTL3-specific shRNA (shR-METTL3) and empty vector, respectively. Transduced cells were selected with puromycin (1 µg/mL) to screen out the stable cell lines at least 7 days before experiments. All related sequences are listed as Additional file 2: Table S1.

**Colony formation, transwell and MTT assays**

The details of cell biology assays were performed as previously described [3].

**Immunohistochemical and immunofluorescence staining**

Immunohistochemical and immunofluorescence staining were performed as previously reported [3] by using the following antibodies: anti-NR4A1 (immunoway), anti-METTL3 (abcam), anti-YTHDF2 (abcam), anti-DDX6 (Saier Biotechnology), anti-DDX6 (Santa Cruze), Ki-67 (CST) and anti-Flag (MBL).

**Direct RNA-seq analysis**

Total RNA was extracted was with TRIzol. Direct RNA-seq analysis was performed at Biomarker Technologies Corporation (Beijing, China).

**Chromatin Immunoprecipitation (ChIP) assay**

The methods of ChIP assay have been described in previous study with minor modification [4]. Briefly, cells were cross-linked with 1% formaldehyde at room temperature for 10 minutes. Then were lysed and sonicated to shear crosslinked DNA to 200-1000 base pairs in length. The sonicated cell lysates were mixed with dilution buffer containing protease inhibitor cocktail and then incubated with Protein G agarose for 1 hour at 4°C with rotation. The recovered supernatant was added indicated immunoprecipitating antibody to incubate for overnight at 4°C.The antibody/chromatin complex was then mixed with protein G agarose at 4°C for 1 hour. After elution, reverse crosslinks and purification, the extracted DNA was analyzed by PCR analysis. The primers used were listed in Additional file 2: Table S1.

**Database analysis**

Several databased or tools were used in this study. The mRNA expression of METTL3, YTHDF2 and NR4A1 in cancer tissues and control non-tumor tissues of were obtained from TCGA (The Cancer Genome Atlas, https://portal.gdc.cancer.gov), GEPIA2 (http://gepia2.cancer-pku.cn/) [5], GEO database (Gene Expression Omnibus, http://www.ncbi.nlm.nih.gov/geo/), GENT2 (http://gent2.appex.kr/gent2/) and TIMER2.0 (http://timer.cistrome.org/) [6]. JASPAR (http://jaspar.genereg.net/) [7], Methprimer(http://www.urogene.org/methprimer/) [8] and QGRS Mapper (https://bioinformatics.ramapo.edu/QGRS/index.php) [9] were preformed to predict the SP1 binding sites of AKT1 promoter. GO (Gene ontology) and KEGG pathway enrichment analysis were performed using online tool of DAVID (http://david.abcc. ncifcrf.gov/). GSEA (Gene set enrichment analysis, http://www.gsea-msigdb.org/gsea/index.jsp) [10] was used to determine the underlying function of NR4A1 and METTL3 in CC. Kaplan-Meier Plotter [11] and GEPIA2 were performed to investigate the prognostic value of METTL3 in CC.

**References**

1. Lin S, Choe J, Du P, Triboulet R, Gregory RI. The m(6)A Methyltransferase METTL3 Promotes Translation in Human Cancer Cells. Mol Cell. 2016;62(3):335-45.

2. Zhu T, Roundtree IA, Wang P, Wang X, Wang L, Sun C, et al. Crystal structure of the YTH domain of YTHDF2 reveals mechanism for recognition of N6-methyladenosine. Cell Res. 2014;24(12):1493-6.

3. Sun Q, Yang Z, Li P, Wang X, Sun L, Wang S, et al. A novel miRNA identified in GRSF1 complex drives the metastasis via the PIK3R3/AKT/NF-κB and TIMP3/MMP9 pathways in cervical cancer cells. Cell Death Dis. 2019;10(9):636.

4. Hu Y, Wu F, Liu Y, Zhao Q, Tang H. DNMT1 recruited by EZH2-mediated silencing of miR-484 contributes to the malignancy of cervical cancer cells through MMP14 and HNF1A. Clin Epigenetics. 2019;11(1):186.

5. Tang Z, Kang B, Li C, Chen T, Zhang Z. GEPIA2: an enhanced web server for large-scale expression profiling and interactive analysis. Nucleic Acids Res. 2019;47(W1):W556-w60.

6. Li T, Fu J, Zeng Z, Cohen D, Li J, Chen Q, et al. TIMER2.0 for analysis of tumor-infiltrating immune cells. Nucleic Acids Res. 2020;48(W1):W509-w14.

7. Fornes O, Castro-Mondragon JA, Khan A, van der Lee R, Zhang X, Richmond PA, et al. JASPAR 2020: update of the open-access database of transcription factor binding profiles. Nucleic Acids Res. 2020;48(D1):D87-d92.

8. Li LC, Dahiya R. MethPrimer: designing primers for methylation PCRs. Bioinformatics. 2002;18(11):1427-31.

9. Kikin O, D'Antonio L, Bagga PS. QGRS Mapper: a web-based server for predicting G-quadruplexes in nucleotide sequences. Nucleic Acids Res. 2006;34(Web Server issue):W676-82.

10. Subramanian A, Tamayo P, Mootha VK, Mukherjee S, Ebert BL, Gillette MA, et al. Gene set enrichment analysis: a knowledge-based approach for interpreting genome-wide expression profiles. Proc Natl Acad Sci U S A. 2005;102(43):15545-50.

11. Lánczky A, Győrffy B. Web-Based Survival Analysis Tool Tailored for Medical Research (KMplot): Development and Implementation. J Med Internet Res. 2021;23(7):e27633.


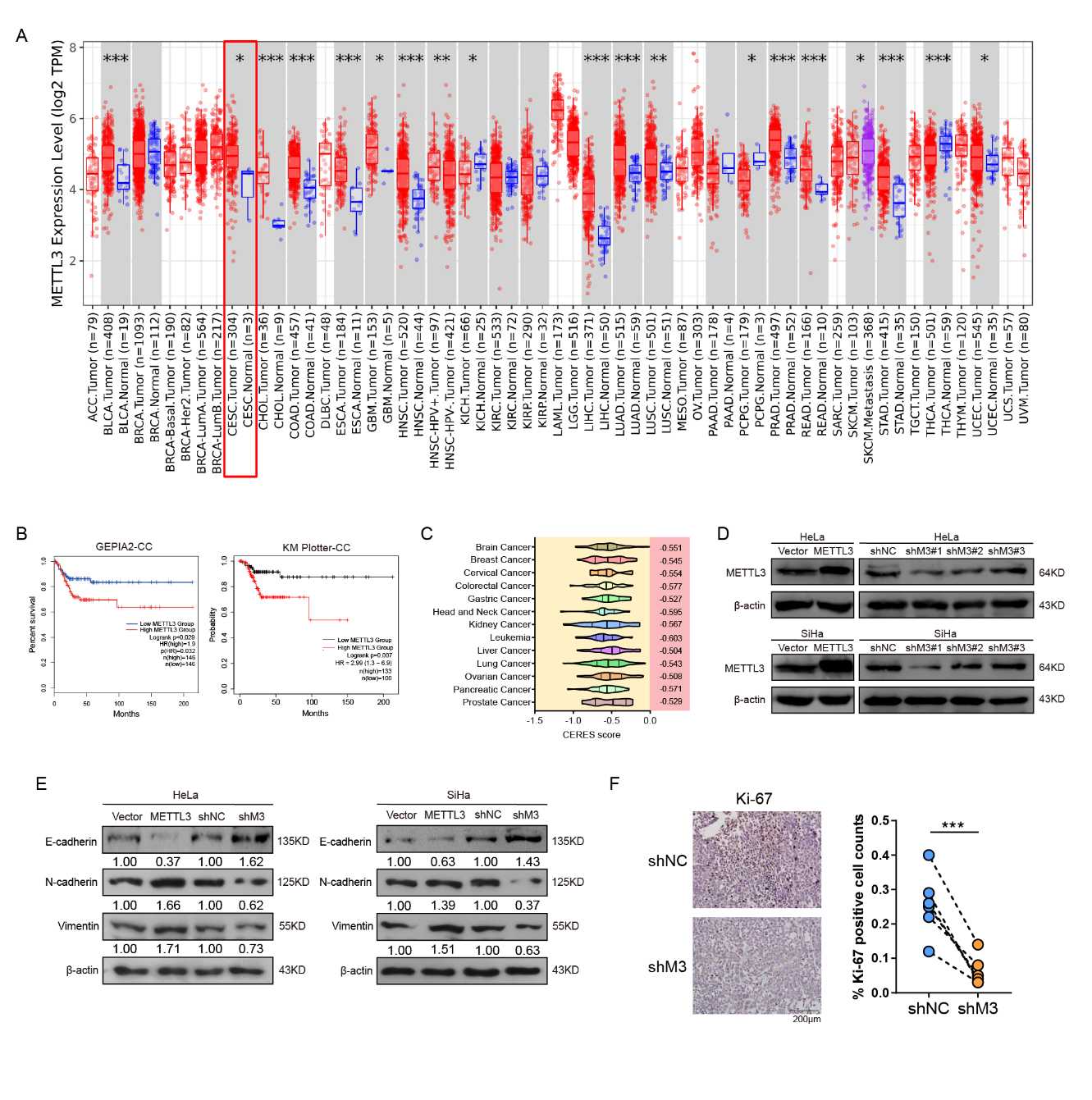
**Supplementary Figures**

**Fig.S1 METTL3 exerts a protumor function in CC.** A, The expression of METTL3 in the vast majority of cancers in TIMER2.0 database. B, The disease-free survival of METTL3 in CC patients from GEPIA2 database (left) and Kaplan-Meier Plotter database (right). C, CERES scores of METTL3 from genome-scale CRISPR–Cas9 essentiality screens. CERES scores of 0 and -1 represent the median effects of nonessential genes and common core essential genes, respectively. For violin plots, midlines indicate the median, upper and lower lines indicate the first and third quartiles. The average of CERES scores for each cancer were also shown. D, Western blot assay was used to verified the efficiency of METTL3 in CC cells. E, The protein levels of N-cadherin, Vimentin and E-cadherin were examined by western blot. F, Representative images of Ki-67 staining of tumor sections in xenograft mouse models (left) and quantitatively analyzed (right). All experiments were performed at least 3 independent times, and data are presented as means ±SD except where otherwise specified. **P*< 0.05, ***P*< 0.01, ****P*< 0.001.


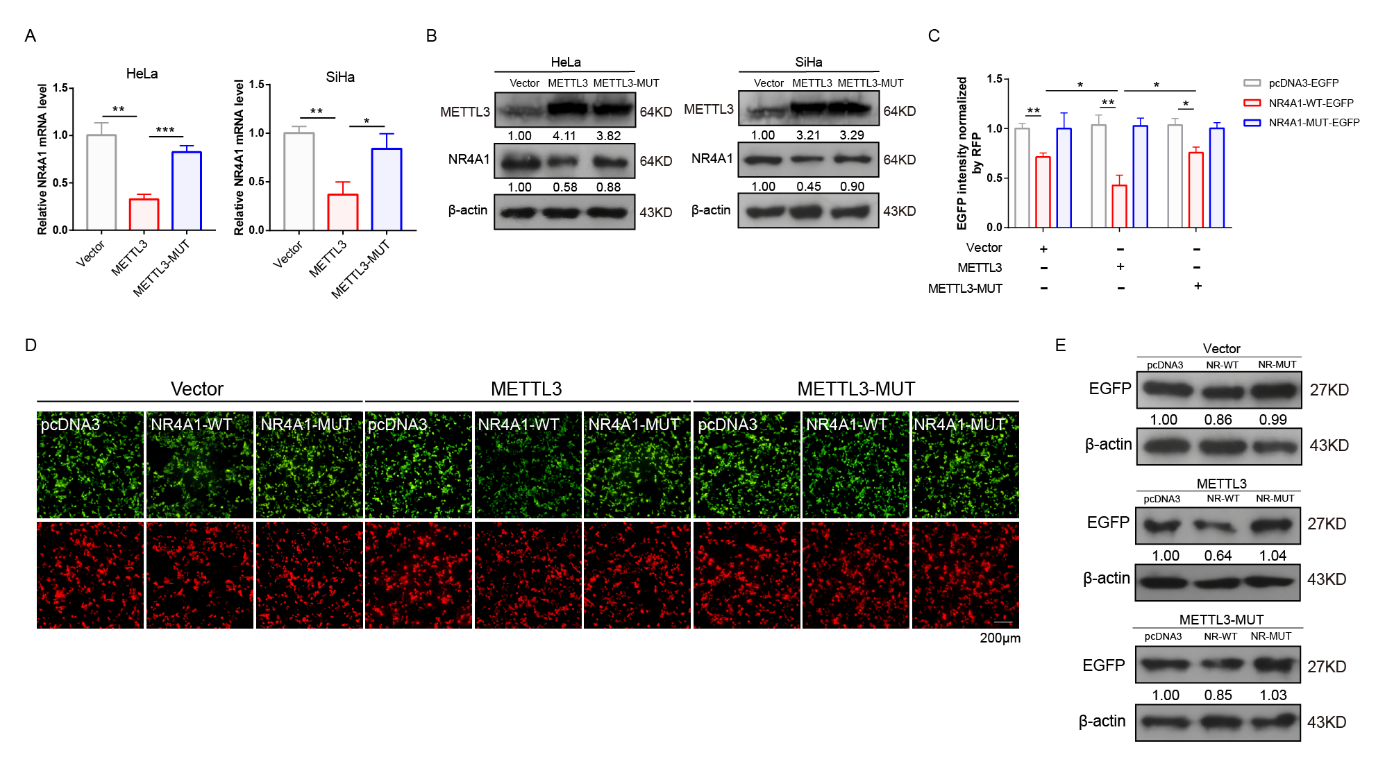


**Fig.S2 METTL3 facilitates NR4A1 mRNA decay through m^6^A modification.**

A-B, The mRNA(A) and protein(B) levels of NR4A1 in CC cells expressing wild-type (WT) or m^6^A recognition defective METTL3 (D395A/W398A). C-D, Representative fluorescence images of relative EGFP activity for cells transfected with the indicated plasmids (D). Quantification of the relative fluorescence intensity (C). RFP was used for normalization. E, EGFP protein levels in cells transfected with the indicated plasmids were measured by western blot analysis. All experiments were performed at least 3 independent times, and data are presented as means ±SD. **P*< 0.05, ***P*< 0.01, ****P*< 0.001.


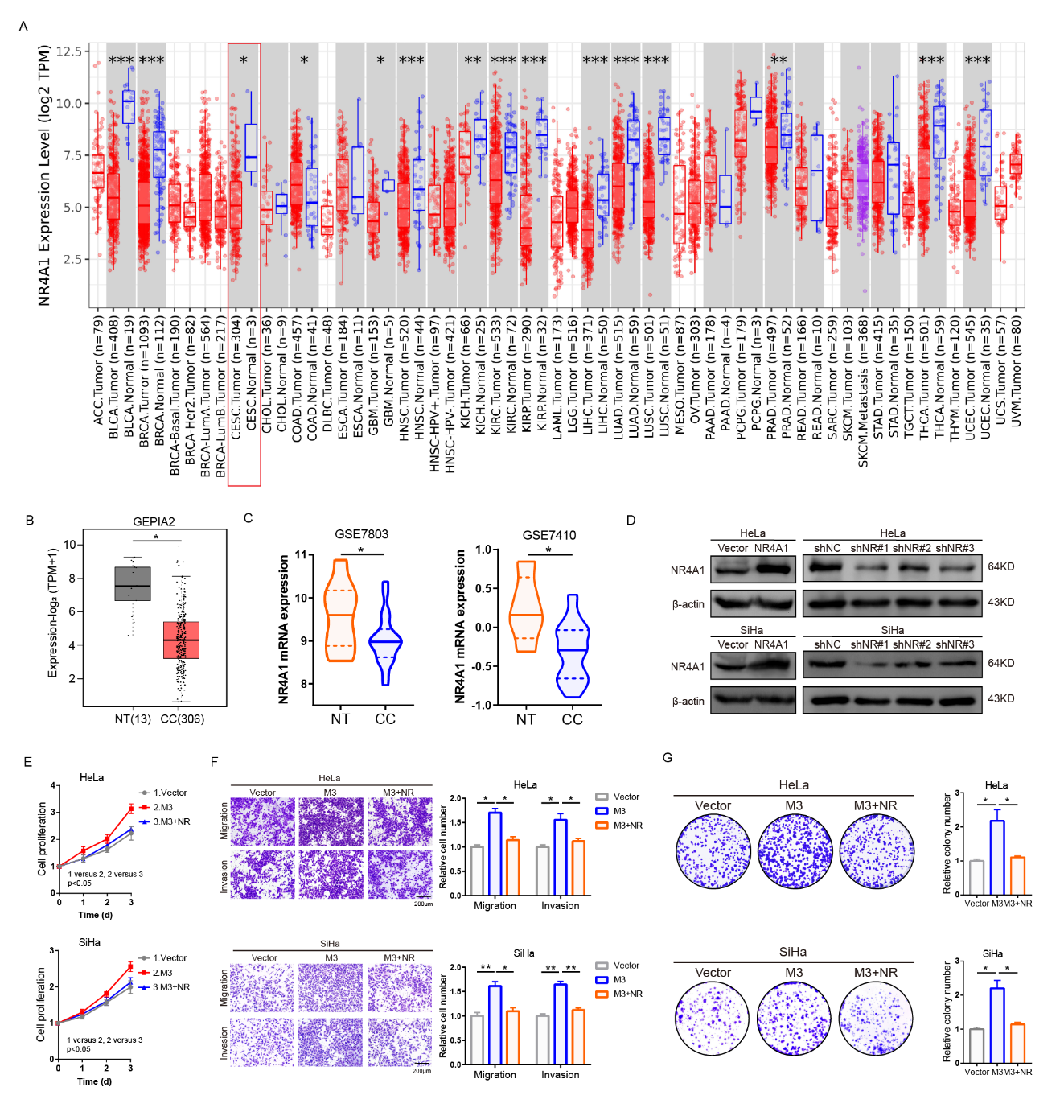


**Fig.S3 NR4A1 inhibits METTL3-induced CC cells progression.**

A-C, The transcriptome data of NR4A1 in CC cohorts of TIMER2.0 (A), GEPIA2 (B) and GEO (C) databases. For violin and box plots, the minimum, first quartile, median, third quartile and the maximum were displayed. D, Western blot assay of NR4A1 levels in overexpression and knockdown of NR4A1 CC cells. E, MTT assay showed the proliferative ability of CC cells by transfected with indicated plasmids. F, The migration and invasion ability of CC cells were measured using transwell assay. G, Colony formation analysis of relative cell proliferation of CC cells transfected with indicated plasmids. All experiments were performed at least 3 independent times, and data are presented as means ±SD except where otherwise specified. **P*< 0.05, ***P*< 0.01, ****P*< 0.001.


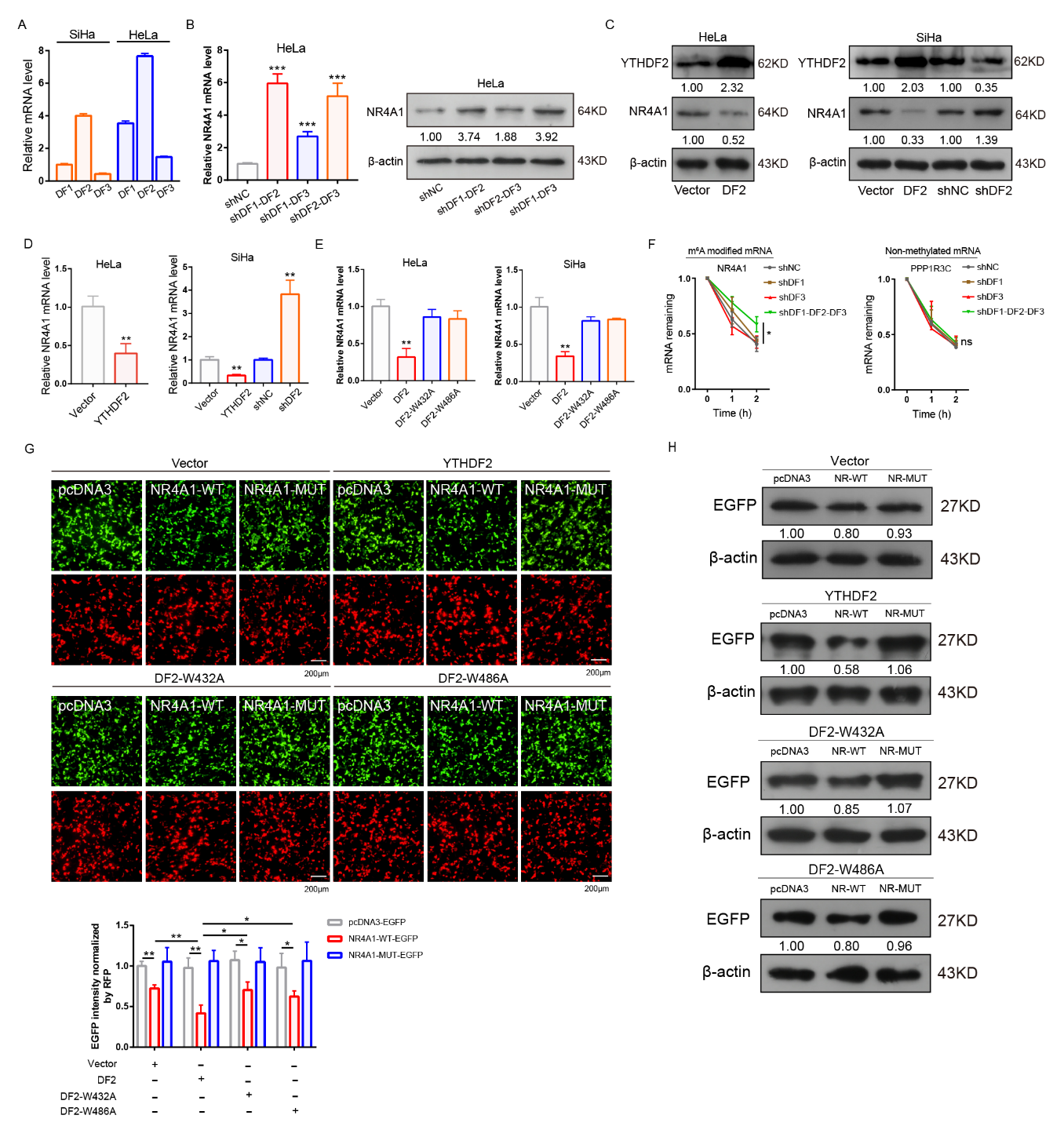


**Fig. S4 YTHDF2 promotes NR4A1 decay through m^6^A-dependent mechanism.**

A, The expression of YTHDF paralogs in CC cells. B, The mRNA (left) and protein (right) levels of NR4A1 after knockdown of the YTHDF paralogs. C-D, The protein (C) and mRNA (D) levels of NR4A1 in overexpression and knockdown of YTHDF2 in CC cells. E, The mRNA levels of NR4A1 in CC cells with overexpression wild-type (WT) or m^6^A recognition defective YTHDF2 (W432A and W486A) plasmids were measured by RT-qPCR. F, The mRNA stability of NR4A1 and non-methylated mRNA (PPP1R3C) upon the silencing of YTHDF paralogs were performed by RT-qPCR after treatment with act-D during indicated time points. G, EGFP reporter assay showed the EGFP activity of co-transfected with the indicated plasmids. RFP was used for normalization. H, The protein levels of EGFP in cells transfected with the indicated plasmids were measured by western blot analysis. All experiments were performed at least 3 independent times, and data are presented as means ±SD. **P*< 0.05, ***P*< 0.01, ****P*< 0.001.


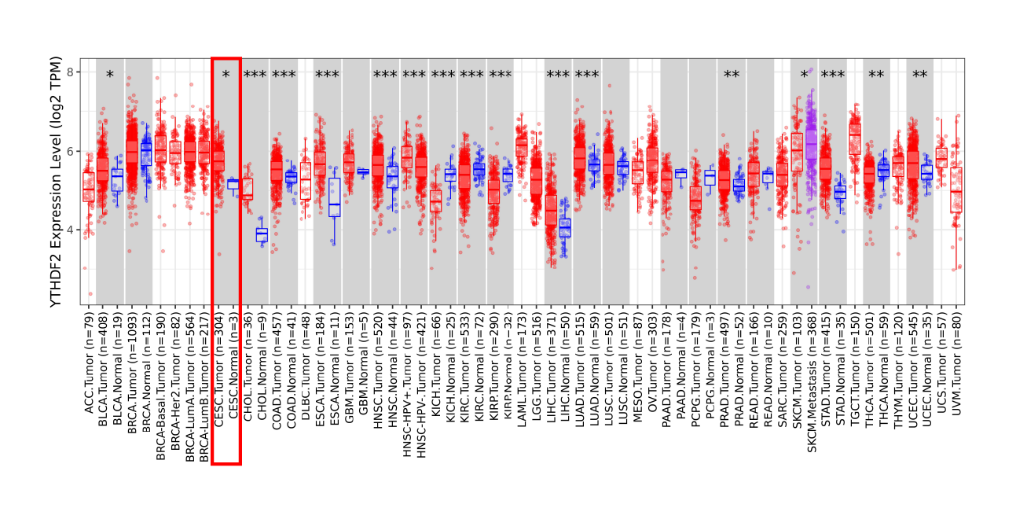


**Fig. S5** TIMER2.0 database of YTHDF2 mRNA in CC tissues.


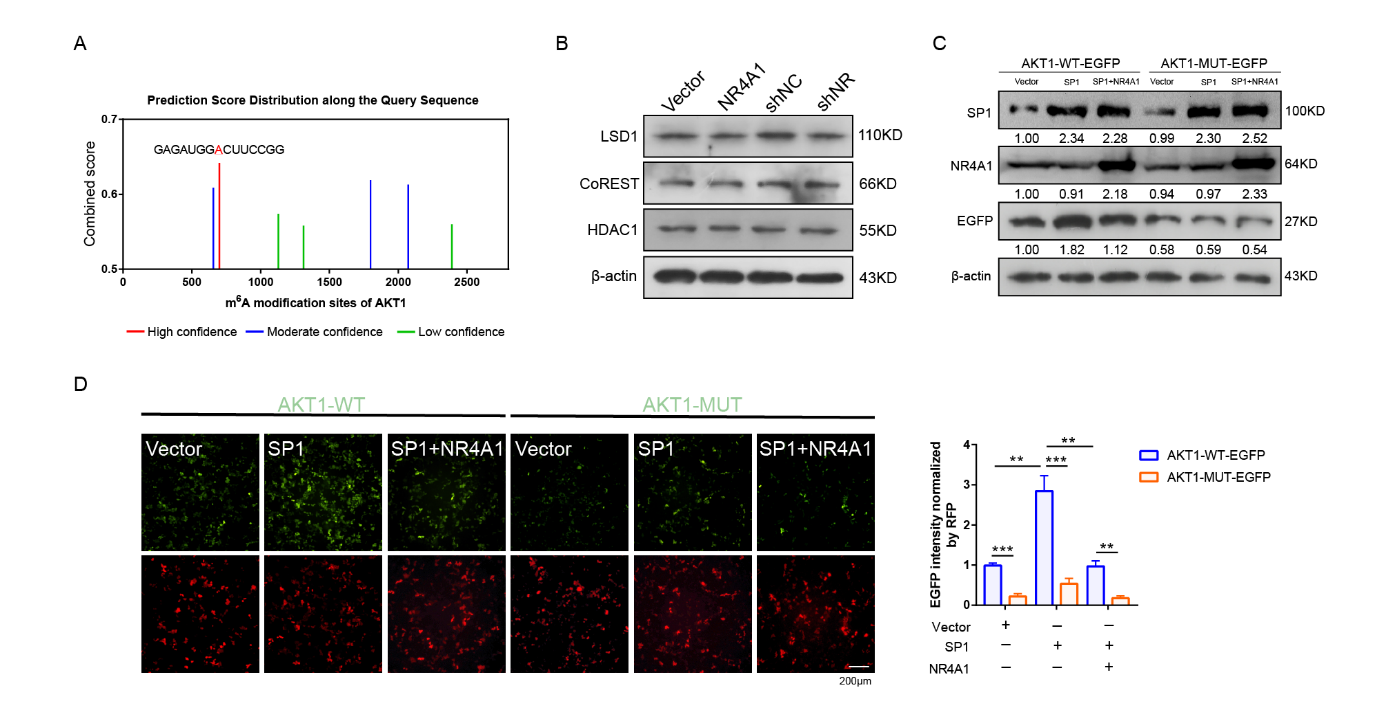


**Fig. S6 YTHDF2-NR4A1 axis promotes transcriptional repression of AKT1 in CC cells.**

A, The potential m^6^A sites of AKT1 were predicted by SRAMP. B, The effect of NR4A1 on the protein levels of each component of LHC complex. C, The protein levels of EGFP were detected by western blot assay upon transfected with indicated plasmids. D, Left: Fluorescent imaging showed that the EGFP intensity after co-transfected with indicated plasmids. Right: Quantification of EGFP activity. RFP were used for normalization. All experiments were performed at least 3 independent times, and data are presented as means ±SD. **P*< 0.05, ***P*< 0.01, ****P*< 0.001.
